# CDK8 Fine-Tunes IL-6 Transcriptional Activities by Limiting STAT3 Resident Time at the Gene Loci

**DOI:** 10.1016/j.celrep.2020.108545

**Published:** 2020-12-22

**Authors:** Jonathan Martinez-Fabregas, Luopin Wang, Elizabeth Pohler, Adeline Cozzani, Stephan Wilmes, Majid Kazemian, Suman Mitra, Ignacio Moraga

**Affiliations:** 1 Division of Cell Signaling and Immunology, School of Life Sciences, University of Dundee, Dundee, UK; 2 Department of Computer Science, Purdue University, West Lafayette, IN, USA; 3 Department of Biochemistry, Purdue University, West Lafayette, IN, USA; 4 Université de Lille, INSERM UMR1277 CNRS UMR9020–CANTHER and Institut pour la Recherche sur le Cancer de Lille (IRCL), Lille, France

## Abstract

Cytokines are highly pleiotropic ligands that regulate the immune response. Here, using interleukin-6 (IL-6) as a model system, we perform detailed phosphoproteomic and transcriptomic studies in human CD4^+^ T helper 1 (Th-1) cells to address the molecular bases defining cytokine functional pleiotropy. We identify CDK8 as a negative regulator of STAT3 transcriptional activities, which interacts with STAT3 upon IL-6 stimulation. Inhibition of CDK8 activity, using specific small molecule inhibitors, reduces the IL-6-induced phosphoproteome by 23% in Th-1 cells, including STAT3 S727 phosphorylation. STAT3 binding to target DNA sites in the genome is increased upon CDK8 inhibition, which results in a concomitant increase in STAT3-mediated transcriptional activity. Importantly, inhibition of CDK8 activity under Th-17 polarizing conditions results in an enhancement of Th-17 differentiation. Our results support a model where CDK8 regulates STAT3 transcriptional processivity by modulation of its gene loci resident time, critically contributing to diversification of IL-6 responses.

## Highlights

• CDK8 regulates IL-6-mediated STAT3 S727 phosphorylation in primary human T cells
• CDK8 controls STAT3 activity by limiting its resident time at gene loci
• CDK8 inhibition increases IL-6-mediated Th17 differentiation

## Graphical Abstract

**Figure:**
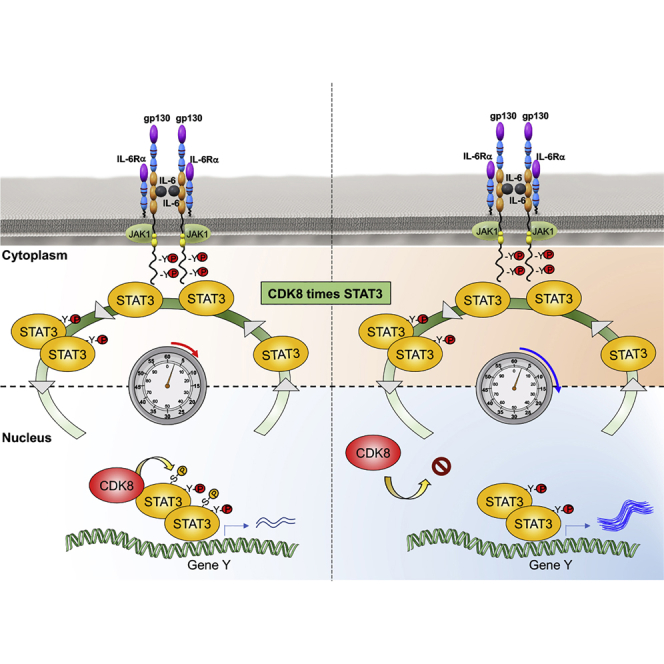

## Teaser

How IL-6 elicits its immune pleiotropic activities is not fully understood. Martinez-Fabregas et al. show that CDK8 represses IL-6-mediated transcription by limiting STAT3 resident time at the gene loci. By regulating CDK8 expression levels, immune cells can adapt their responses to STAT3-activating cytokines.

## Introduction

Cytokines are critical orchestrators of innate and adaptive immunity (Lin and Leonard, 2019). Despite the functional relevancy of this family of ligands, the molecular basis governing their large functional pleiotropy remains poorly defined. Cytokines exert their activities by dimerizing/oligomerizing surface receptors and triggering the tyrosine (Tyr) phosphorylation of STAT transcription factors by janus kinases (JAKs) (Gorby et al., 2018; Martinez-Fabregas et al., 2019; Stroud and Wells, 2004; Wang et al., 2009; Wilmes et al., 2020). This in turn leads to the nuclear translocation of STATs and the induction of specific gene expression programs and bioactivities (Poli and Camporeale, 2015; Schindler et al., 2007). However, how qualitative and quantitative changes in these pathways contribute to cytokine functional pleiotropy is poorly understood.

STATs can be modified in conserved Tyr or serine (Ser) residues (Decker and Kovarik, 2000). Although STAT Tyr phosphorylation plays a critical role in mediating cytokine responses, the role of STAT Ser phosphorylation in cytokine-mediated activities is less clear (Chung et al., 1997; Decker and Kovarik, 2000; Kim and Baumann, 1997; Wen et al., 1995). Early work in cancer cell lines showed that Ser phosphorylation of STAT proteins regulated their transcriptional activities (Wen et al., 1995). However, whether STAT Ser phosphorylation promotes a positive or negative effect on STAT transcriptional activities remains more controversial (Bancerek et al., 2013; Decker and Kovarik, 2000; Levy and Darnell, 2002; Lim and Cao, 1999). Some studies report a positive effect of STAT Ser phosphorylation in driving STAT transcriptional activities, whereas others have reported an opposite effect (Bancerek et al., 2013; Decker and Kovarik, 2000; Levy and Darnell, 2002; Lim and Cao, 1999; Steen et al., 2016; Wen et al., 1995; Yokogami et al., 2000). For STAT3, Ser phosphorylation appears to negatively impact its transcriptional activities by regulating its chromatin binding dwell time (Yang et al., 2020). But, earlier studies had reported that STAT3 Ser phosphorylation did not have effects on STAT3 chromatin binding (Wen and Darnell, 1997). The differences in these observations could be attributed to the use of different cancer cell lines, suggesting a context-dependent regulation of STAT3 signaling.

Interleukin-6 (IL-6) represents a classical paradigm for cytokine functional pleiotropy. IL-6 acts as a central regulator of the immune response by triggering both pro-inflammatory and anti-inflammatory responses (Hunter and Jones, 2015; O’Shea and Murray, 2008; Rose-John, 2018; Scheller et al., 2011). IL-6 drives inflammatory processes by modulating the adaptive and innate immunity arms. On the one hand, IL-6 promotes the differentiation of T helper-17 (Th-17) cells, while inhibiting the differentiation of T regulatory (T reg) cells (Jones et al., 2010; Korn et al., 2008). On the other hand, IL-6 recruits myeloid cells to sites of inflammation (Fielding et al., 2008; Gabay, 2006). Additionally, dysregulation of IL-6 or IL-6-mediated responses is often associated with inflammatory disorders, making this cytokine highly relevant for human health (Jones and Jenkins, 2018; Tanaka et al., 2014). IL-6 exerts its activities by triggering the activation of the JAK1/STAT1/STAT3 signaling pathway upon recruitment of IL-6Rα and gp130 receptor subunits (Heinrich et al., 1998; Martinez-Fabregas et al., 2019; Servais et al., 2019). However, despite the critical contribution that IL-6 plays in modulating the immune response, the role that STAT3 Ser phosphorylation plays on regulating IL-6 immune activities has not been explored in detail. Studies on STAT1 have shown that CDK8 appears to be the kinase driving its Ser phosphorylation in response to interferon gamma (IFNγ) stimulation in macrophages (Bancerek et al., 2013). Blockage of STAT1 Ser phosphorylation, using STAT1 Ser mutants, significantly altered the IFNγ transcriptional response and its ability to clear *Listeria monocytogenes* infection, highlighting the relevance of STAT1 Ser phosphorylation in modulating IFNγ response in macrophages (Varinou et al., 2003). However, how Ser phosphorylation fine-tunes STAT3 immuno-modulatory activities is less well known.

In this study, we set out to characterize how the signaling initiated by IL-6 in human T cells leads to its functional pleiotropy. We detected both STAT3 Tyr and Ser phosphorylations in response to IL-6 stimulation, with STAT3 Ser phosphorylation exhibiting a delayed activation. Using a battery of small molecule inhibitors, we identified CDK8 and CDK9 as the kinases driving Ser phosphorylation of STAT3 upon IL-6 stimulation in T cells. Using proximity ligation studies, we confirmed the increased interaction between STAT3 and CDK8 and CDK9 in the nucleus upon IL-6 stimulation. Inhibition of the activity of these two kinases resulted in a more robust interaction with STAT3, even in the absence of IL-6 stimulation. Chromatin immunoprecipitation sequencing (ChIP-seq) and RNA sequencing (RNA-seq) studies revealed a global increase in STAT3 chromatin binding upon inhibition of CDK8, resulting in the induction of a larger gene expression program by IL-6. In agreement with this enhanced gene expression program, IL-6 induced a more robust differentiation of Th-17 cells upon CDK8 inhibition *in vitro*. Overall, our studies identify a STAT3 regulatory mechanism in T cells, whereby CDK8 and CDK9 modulate STAT3 processivity by controlling its chromatin binding dwell time and transcriptional activity. These observations suggest ways to manipulate IL-6- and STAT3-mediated responses by fine-tuning CDK8/9 activities.

## Results

### IL-6 Signaling Preferentially Induces Tyr/Ser Phosphorylation of STAT1/3 in Human T Cells

**Figure 1:**
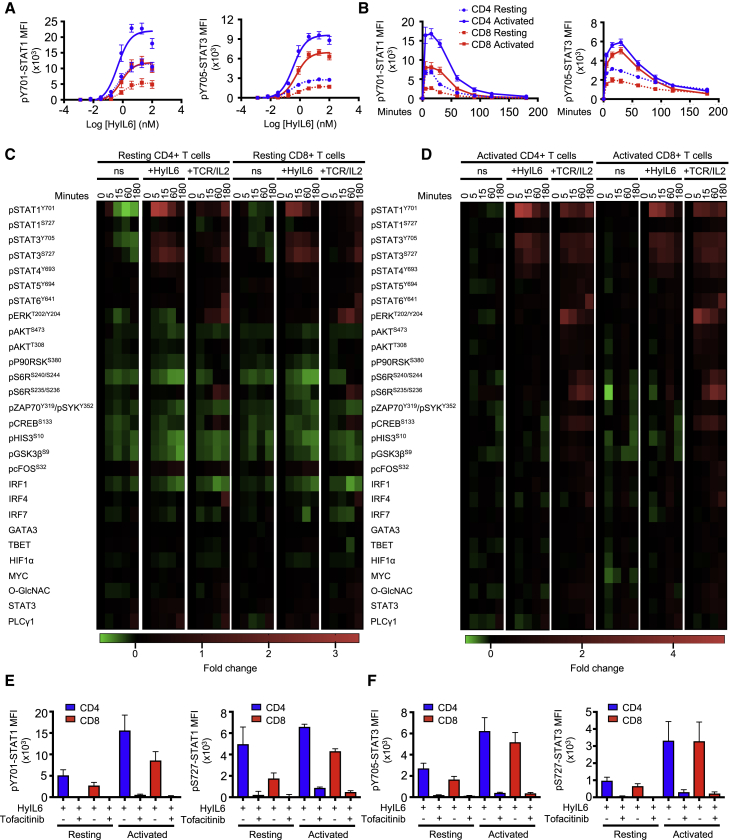
IL-6 Signaling Landscape in Primary Human T Cells (A and B) STAT1 and STAT3 phosphorylation in response to various doses (A) and exposure time (B) of IL-6 stimulation in resting and activated primary human CD4^+^ and CD8^+^ T cells. Error bars show mean ± SEM from three individual biological replicas. (C and D) Phospho-FLOW analysis of IL-6 signaling pathways in resting (C) and activated primary human CD4^+^ and CD8^+^ T cells treated with HyIL-6 or anti-CD3/CD28 (TCR) + IL-2. ns, cells without any stimulation. Heatmaps show fold change in the level of phosphorylation or protein expression of the different proteins. See also Figures S2 and S3. (E and F) Effect of JAK inhibition (2 μM tofacitinib) on the phosphorylation of STAT1 (E) and STAT3 (F) Tyr701 and Ser727 in resting and activated primary human CD4^+^ and CD8^+^ T cells. Error bars show mean ± SEM from three individual biological replicas.

IL-6 critically contributes to modulating the T cell response. Yet, we have a poor understanding of the signaling networks engaged by IL-6 in T cells and their role in shaping IL-6 immune activities. To gain insight into the IL-6 signalosome in T cells, we carried out detailed signaling studies in human resting or activated CD4^+^ and CD8^+^ T cells stimulated with IL-6. IL-6 receptor expression varies significantly among different T cell populations and environmental contexts (Figure S1; Betz and Müller, 1998; Jones et al., 2010; Ridgley et al., 2019), making the study of IL-6 signaling in T cells challenging. To minimize this variability, we have used Hyper-IL-6 (HyIL-6) for our signaling studies. HyIL-6 is a synthetic heterodimer comprised of IL-6Rα and IL-6 proteins connected by a gly/ser linker (Fischer et al., 1997). HyIL-6 triggers signaling in all cells expressing gp130, producing a more robust and homogeneous signaling output (Rose-John, 2012). In Th-1 cells, HyIL-6 induced a more potent phosphorylation of STAT1 and STAT3 than IL-6 (Figure S1A, left panels). However, the pSTAT1/pSTAT3 ratio induced by the two ligands was identical (Figure S1A, right panel), suggesting that HyIL-6 exhibits only qualitative and no quantitative signaling differences with IL-6. Dose-response (Figure 1A) and kinetic signaling studies (Figure 1B) showed that resting and activated CD4^+^ and CD8^+^ T cells respond to HyIL-6 treatment by Tyr phosphorylating STAT1 and STAT3 transcription factors. The STAT1/3 activation amplitudes elicited by HyIL-6 in these cells, however, differ significantly, with activated CD4^+^ and CD8^+^ T cells triggering between 2- to 3-fold higher STAT1/3 phosphorylation amplitudes than resting CD4^+^/CD8^+^ T cells in response to HyIL-6 treatment (Figures 1A and 1B). Interestingly, activated CD4^+^ T cells triggered higher levels of STAT1 phosphorylation than activated CD8^+^ T cells upon HyIL-6 stimulation (Figures 1A and 1B, left panels), which correlated with a higher expression of gp130 and IL-6Rα by CD4^+^ T cells (Figure S1B). Overall, these results show that IL-6-induced signaling in human T cells is a dynamic and context-dependent process.

To gain further insight into the signaling networks, beyond JAK/STAT1/3, engaged by IL-6 in T cells, we used an antibody array targeting 28 relevant signaling intermediaries. Resting and activated CD4^+^ and CD8^+^ T cells were stimulated with saturating concentrations of HyIL-6 for the indicated times, and their signaling signatures in response to HyIL-6 treatment were assayed by flow cytometry (Figures 1C and 1D). To ensure the quality of our signaling antibody array, we stimulated different populations of T cells with anti-CD3/anti-CD28 antibodies (TCR)+IL-2 as a positive control, because this treatment activates a large proportion of the signaling molecules detected by our antibody array (Ross et al., 2016; Smith-Garvin et al., 2009). In both resting and activated human CD4^+^ and CD8^+^ T cells, TCR+IL-2 treatment led to the activation of a large proportion of the signaling intermediaries, including STAT1, STAT3, STAT4, STAT5, STAT6, ERK, AKT, S6R, and CREB (Figures 1C and 1D; Figures S2 and S3). HyIL-6 treatment on the other hand preferentially induced the Tyr and Ser phosphorylation of both STAT1 and STAT3 and, to a lower extent, STAT4 (Figures 1C and 1D; Figure S2). STAT1 and STAT3 Tyr and Ser phosphorylation in response to HyIL-6 treatment were inhibited by the JAK inhibitor tofacitinib, confirming the dependency of these two modifications on JAK activity (Figures 1E and 1F; Figures S3A–S3D). Overall, our signaling data support a dynamic activation of the JAK/STAT pathway by IL-6 in T cells, suggesting that IL-6 functional pleiotropy emanates from activation of a few signaling intermediaries.

### IL-6 Induces a Large Number of Phosphoproteome Changes in Human T Cells

**Figure 2:**
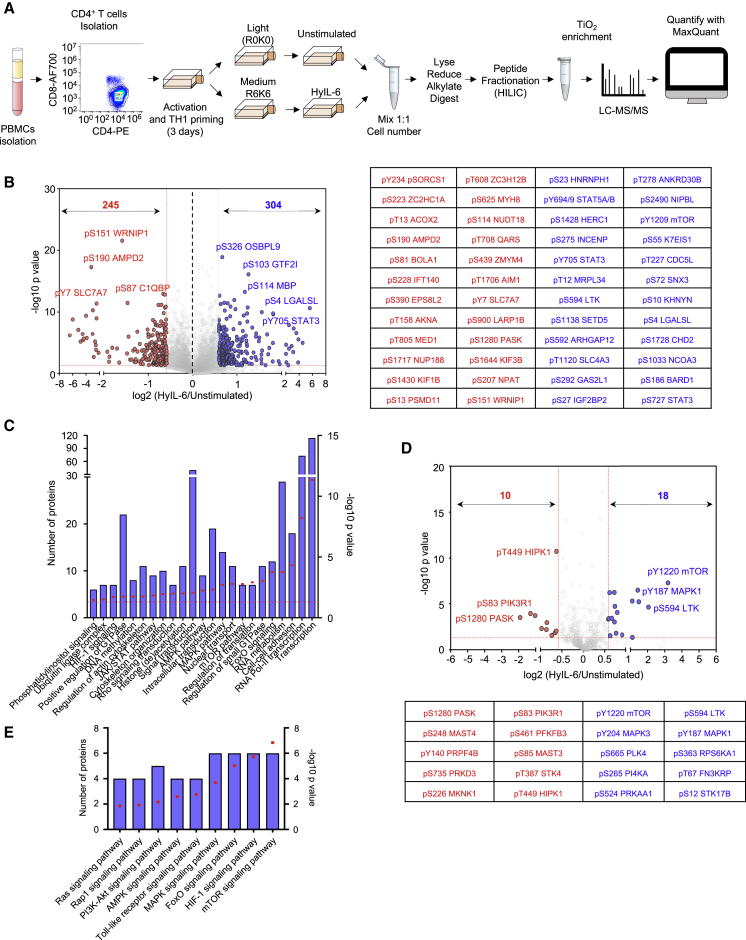
Phosphoproteomic Landscape of IL-6 in Human Primary CD4^+^ Th-1 Cells (A) Experimental workflow for SILAC-based quantitative phosphoproteomic analysis of human primary CD4^+^ Th-1 cells stimulated with 20 nM HyIL-6 for 15 min. (B) Volcano plot showing differential phosphopeptides in unstimulated versus stimulated Th-1 cells with 20 nM HyIL-6 for 15 min. Phosphopeptides identified in six biological replicates are shown as log-transformed SILAC ratios plotted against log-transformed p values (two-sided t test). Phosphosites changed more than 1.5-fold with a p value of <0.05 are shown in red (decreased) or blue (increased). Select phosphopeptides are labeled (see also Table S1 for full list). The 24 phosphosites more reproducibly decreased (red) or increased (blue) are displayed alongside. (C) Gene Ontology (GO) analysis as determined by DAVID of the phosphosites regulated by HyIL-6 in human primary Th-1 cells. (D) Kinase phosphosites regulated in response to HyIL6 stimulation in human primary Th-1 cells. (E) GO analysis as determined by DAVID showing main signaling pathways engaged by HyIL-6 in human primary Th-1 cells.

To obtain a full spectrum of the IL-6 signalosome in T cells, we next performed a quantitative high-resolution phosphoproteomics assay using stable isotope labeling by amino acids in cell (SILAC). We selected Th-1 cells due to their significance in immunity and ability to expand *in vitro* to large quantities of a highly pure population, which is compatible with SILAC studies (Figure 2A).

The combined analysis from six independent biological replicates of unstimulated versus HyIL-6-stimulated Th-1 cells identified 17,935 phosphosites on 4,196 proteins (Figure 2B; Table S1). Among those phosphosites, 304 were increased and 245 were decreased significantly in response to HyIL-6 stimulation in human primary Th-1 cells, while the rest remained unchanged (Figure 2B). Gene Ontology (GO) analysis (KEGG, Kyoto Encyclopedia of Genes and Genomes) pathways and molecular function analyses) showed an enrichment of the JAK/STAT pathway, as expected, upon stimulation of Th-1 cells with HyIL-6 (Figure 2C). Of all proteins with detected phosphosites, 288 were kinases (Figure 2D; Table S1). Of those proteins, 28 were regulated by HyIL-6 treatment (Figure 2D; Table S1) and were enriched in several signaling pathways (e.g., mTOR and mitogen-activated protein kinase [MAPK]) (Figure 2E). GO analysis showed that 41% of the phosphoproteomic changes induced by HyIL-6 took place in the nucleus (Figures S3B and S4), highlighting this compartment as an important signaling platform for IL-6 activities. Furthermore, our GO analysis indicated an enrichment of phosphosites involved in the regulation of transcription and more specifically RNA polymerase II (RNA Pol II)-mediated transcription (Figure 2C). HyIL-6 treatment regulated processes related to protein transcription, acting as part of DNA-modifying complexes (MTA1, CCAR2, and TRRAP) or serving as transcriptional regulators (ELF2, RUNX2, TSC22D4, and SP4); histone (de)acetylation (KANSL2, TRRAP, and RBBP7); RNA Pol II transcription (MECPE, MEF2C, and MED1); and mRNA splicing (DDX46 and SF3B4), processing (RBM6 and RBM39), and export (NUP50 and NUP153) (Figures S3B and S4). HyIL-6 treatment also regulated non-nuclear processes, including regulation of the cytoskeleton, translation, and proteasome at the cytoplasmic level (Figure 2C; Figures S3 and S4). Overall, our phosphoproteomic data revealed a strong regulation of the nuclear phosphoproteome by IL-6 that could contribute to fine-tuning its immuno-modulatory activities.

### CDK8/CDK9 Regulate STAT1 and STAT3 Ser727 Phosphorylation

**Figure 3:**
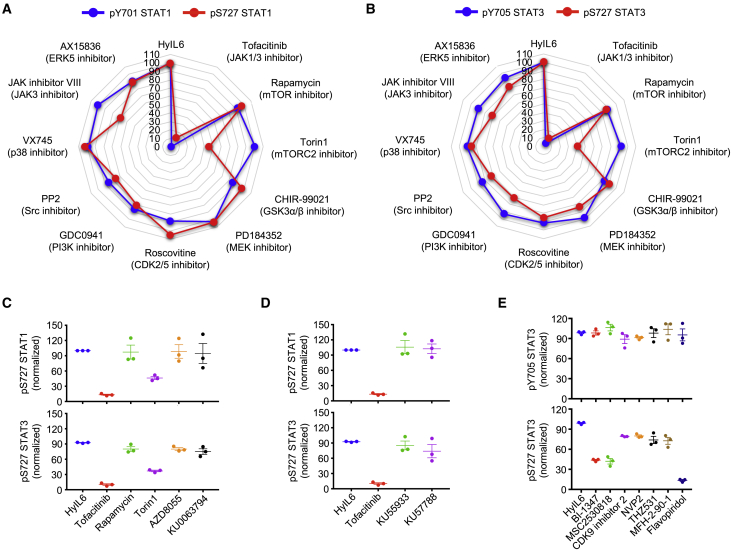
STAT1 and STAT3 HyIL-6-Induced Ser727 Phosphorylation Is CDK8/9 Mediated (A and B) Spider plots showing pTyr701 STAT1 (A) or pTyr705 STAT3 (B) (blue line) and pSer727 STAT1 (A) or pSer727 STAT3 (B) (red line) MFI normalized to HyIL-6-treated cells in the presence of different inhibitors in human primary CD4^+^ Th-1 cells. (C) Effect of different mTOR inhibitors on the STAT1 (top panel) and STAT3 (bottom panel) Ser727 phosphorylation induced by HyIL-6 in human primary CD4^+^ T cells. (D) Effect of ATM inhibitor (KU53933) and DNA-PK inhibitor (KU57788) on the STAT1 (top panel) and STAT3 (bottom panel) Ser727 phosphorylation induced by HyIL-6 in human primary CD4^+^ T cells. (E) Effect of different CDK inhibitors on the STAT3 Tyr705 (top panel) and STAT3 Ser727 (bottom panel) phosphorylation induced by HyIL-6 in human primary CD4^+^ T cells. For all experiments, quantitative data were calculated from three individual biological replicates. Error bars show mean ± SEM.

HyIL-6 triggers the Tyr and Ser phosphorylation of STAT1 and STAT3 in T cells. Although JAK1 contributes to the Tyr phosphorylation of STAT1/3, the kinase responsible for STAT1/3 Ser phosphorylation in T cells is currently not known. To identify this kinase, we used a panel of inhibitors targeting signaling pathways previously shown to regulate STAT1 and STAT3 Ser phosphorylation in different cellular systems (Decker and Kovarik, 2000). Tofacitinib, a JAK inhibitor, blocked both STAT1/STAT3 Tyr and Ser phosphorylation by HyIL-6 treatment (Figures 3A and 3B), confirming previous observations. Of the battery of inhibitors tested, only Torin 1, an mTOR inhibitor targeting both mTORC1 and mTORC2 complexes (Thoreen et al., 2009), inhibited the Ser phosphorylation induced by HyIL-6 in both STAT1 and STAT3, without affecting their Tyr phosphorylation (Figures 3A and 3B). Rapamycin, an inhibitor that under our experimental conditions only targets the mTORC1 complex (Laplante and Sabatini, 2012; Sarbassov et al., 2006), failed to do so, suggesting that the Ser phosphorylation of STAT1 and STAT3 induced by HyIL-6 was a mTORC2-mediated response (Figures 3A and 3B). However, alternative mTOR inhibitors (i.e., AZD8055 and KU0063794) failed to restrict STAT1 and STAT3 Ser phosphorylation by HyIL-6, indicating that the Torin1-mediated inhibition was an off-target effect (Figure 3C). Moreover, inhibitors specifically targeting well-described off-targets of Torin 1, i.e., ataxia telangiectasia mutated (ATM; KU53933) and DNA-dependent protein kinase (DNA-PK; KU57788) (Liu et al., 2012), failed to inhibit STAT1 and STAT3 Ser phosphorylation by HyIL-6 (Figure 3D). This finding indicates that the inhibition of STAT1 and STAT3 Ser phosphorylation is a previously unknown off-target effect of Torin1 and needs to be accounted for when used in *in vitro* or *in vivo* studies.

Previous studies have described CDKs as regulators of STAT1 Ser phosphorylation in different systems (Bancerek et al., 2013; Chen et al., 2019; Kosciuczuk et al., 2019; Putz et al., 2013). Thus, we next tested whether the STAT3 Ser phosphorylation induced by HyIL-6 was mediated by CDKs. For that test, we measured STAT3 Tyr and Ser phosphorylation levels induced by HyIL-6 in cells treated with a panel of CDK inhibitors. Flavopiridol, a well-described pan-CDK inhibitor (Luke et al., 2012), completely abolished the Ser phosphorylation of STAT3 induced by HyIL-6 (Figure 3E; Figures S5A–S5C). CDK8-specific inhibitors (i.e., BI-1347 in Hofmann et al., 2020 and MSC2530818) inhibited HyIL-6 induced STAT3 Ser phosphorylation by 70% in both human CD4^+^ and CD8^+^ T cells (Figure 3E; Figures S5A–S5C). CDK9 (i.e., NVP2 and CDK inhibitor II) or CDK12/CDK13 (i.e., THZ531 and MFH-2-90-1) inhibitors only reduced the STAT3 Ser phosphorylation by 20% (Figure 3E; Figures S5A–S5C). None of the inhibitors affected the STAT3 Tyr phosphorylation levels (Figure 3E, Figures S5A–S5C). Genetic silencing of CDK8 and CDK9 in HEK293T cells (Figures S5D–S5F) and *in vitro* kinase assays performed with recombinant CDK7, CDK8, CDK9, and STAT3 proteins (Figure S5G) further support CDK8 and CDK9 as the main kinases driving STAT3 Ser phosphorylation in response to HyIL-6. We detected a small decrease on STAT3 Tyr phosphorylation upon HyIL-6 stimulation in cells depleted of CDK8 and CDK9 (Figure S5E), which we did not detect in experiments using small molecule inhibitors. We believe that this decrease results from toxicity associated with prolonged depletion of CDK8 and CDK9 kinases. Our data highlight a critical role of CDK8 in regulating STAT3 Ser phosphorylation by HyIL-6, with an accessory role of other CDK members.

### HyIL-6 Induces Nuclear Interaction of STAT3 and CDK8/CDK9

**Figure 4:**
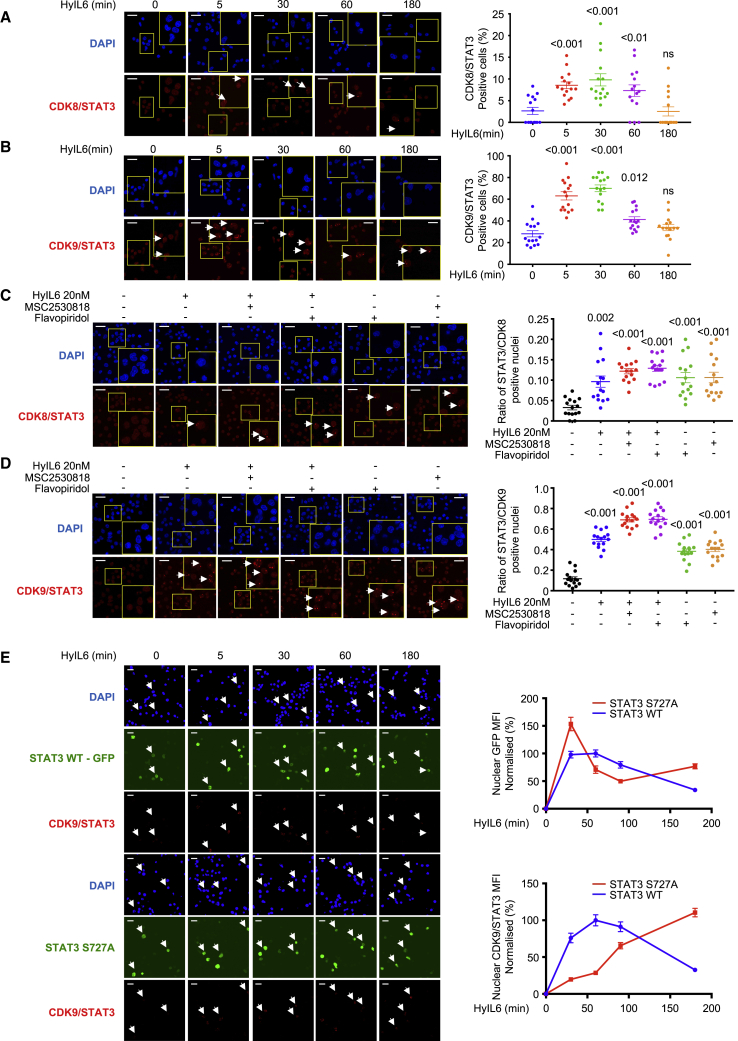
PLA Analysis of the Interaction of STAT3 and CDK8/9 Induced upon HyIL-6 Stimulation in Human Primary CD4^+^ Th-1 Cells (A and B) Kinetics of the STAT3/CDK8 (A) or STAT3/CDK9 (B) interaction induced by 20 nM HyIL-6 in human primary CD4^+^ Th-1 cells. Scale bars, 20 μm. Statistical significance was calculated by one-way ANOVA. (C and D) STAT3/CDK8 (C) or STAT3/CDK9 (D) interactions were analyzed by PLA upon 20 nM HyIL-6 stimulation in the absence or presence of 2 μM MSC2530818 or 2 μM flavopiridol or upon treatment with the inhibitor only. Scale bars, 20 μm. Statistical significance was calculated by unpaired t test. White arrows in A to D indicate examples of cells where interaction signal was detected. Cumulative plots from n = 15 pictures alongside show the percentage of positive cells. Error bars show mean ± SEM. The p values were calculated based on non-parametric two-tailed Wilcoxon rank-sum test against the control group (first bar on the left). (E) STAT3/CDK9 interaction analyzed by PLA upon 20 nM HyIL-6 stimulation in STAT3 KnD Hut78 cells reconstituted with STAT3 WT-GFP (top panel) or STAT3 S727A-GFP (bottom panels). White arrows indicate examples of cells expressing the recombinant protein and where the STAT3/CDK9 interaction was detected by PLA. Scale bars, 20 μm. Graphs alongside show the nuclear GFP MFI normalized to unstimulated cells (top graph) or the nuclear STAT3/CDK9 PLA MFI in GFP-positive cells normalized to unstimulated cells (bottom graph). Quantitative data generated from n = 15 pictures. Error bars show mean ± SEM.

Next, we explored whether STAT3 and CDK8/9 physically interacted in the nucleus upon HyIL-6 stimulation. For that, we performed proximity ligation assays (PLAs), a technique that allows the detection of protein complexes at endogenous levels without the need of protein overexpression or labeling that could interfere with their binding partners (Fredriksson et al., 2002). Activated human primary CD4^+^ T cells were stimulated with 20 nM HyIL-6 for the indicated times, and samples were prepared for PLA analysis following manufacturer instructions (Sigma). In untreated cells, we detected very low levels of STAT3/CDK8 (Figure 4A) and STAT3/CDK9 complexes (Figure 4B). Upon HyIL-6 stimulation, we detected a 2- to 4-fold increase in the number of STAT3/CDK8 and STAT3/CDK9 complexes, which peak at 30 min after stimulation and return to basal levels after 3 h (Figures 4A and 4B), paralleling the STAT3 Tyr activation kinetics (Figure 1B).

We next studied whether CDK activity modulated the formation of STAT3/CDK complexes upon HyIL-6 stimulation. Activated human CD4^+^ T cells were stimulated with HyIL-6 for 30 min in the presence of different CDKs inhibitors. Levels of STAT3/CDK8 and STAT3/CDK9 complexes were measured by PLA analysis. As before, HyIL-6 stimulation led to a significant increase in the number of STAT3/CDK8 and STAT3/CDK9 complexes, when compared to unstimulated cells (Figures 4C and 4D). Addition of flavopiridol (panCDK inh.) or MSC2530818 (CDK8 inh.) inhibitors resulted in an enhancement in the number of STAT3/CDK8 and STAT3/CDK9 complexes (Figures 4C and 4D). Interestingly, treatment with the two inhibitors led to an increase in the number of STAT3/CDK8 and STAT3/CDK9 complexes (Figures 4C and 4D) in the absence of HyIL-6 stimulation, suggesting a role for CDK activities in regulating STAT3 nuclear resident time and thus chromatin binding.

Previous studies have reported that mutation of S727 in STAT3 to alanine (Ala) modulates its transcriptional activity (Chung et al., 1997; Kim and Baumann, 1997; Lim and Cao, 1999; Wen et al., 1995; Yokogami et al., 2000). Thus, we next investigated the role that this mutation plays on recruitment of CDKs upon HyIL-6 stimulation. For that investigation, we took advantage of the human Hut78 cell line, a cutaneous T lymphocyte, where HyIL-6 treatment also induced STAT3 S727 phosphorylation in a CDK-dependent manner (Figure S6A-B). Importantly, only flavopiridol treatment resulted in an inhibition of STAT3 Ser phosphorylation by HyIL-6 in Hut78 cells, suggesting that in these cells, CDK9 but not CDK8 is the main CDK driving STAT3 phosphorylation (Figures S6A and S6B). Next, we generated STAT3 knockdown (STAT3 KnD) Hut78 cell lines by CRISPR-Cas9 (Figure S6C). These cells exhibited a clear reduction in the STAT3 Tyr phosphorylation upon HyIL-6 stimulation (Figure S6D). STAT3 KnD cells were reconstituted with wildtype (WT) STAT3-GFP or S727A STAT3-GFP mutant, and the levels of STAT3/CDK9 complex formation were measured by PLA (Figure 4E; Figure S6E). Due to STAT3 overexpression in these cells, we detected significantly higher levels of the STAT3/CDK9 complex in unstimulated cells than those detected in human Th-1 cells. Yet, upon HyIL-6 stimulation, we observed a significant increase in the number of STAT3 WT/CDK9 complexes in the nucleus, which peaked at 30 min and went back to basal levels by 2 h after treatment (Figure 4E). The STAT3 S727A mutant exhibited a similar nuclear translocation profile to STAT3 WT, but it showed a delayed association kinetic with CDK9 (Figure 4E). Interestingly, at late stimulation times, we observed higher levels of STAT3 S727A/CDK9 complexes when compared to STAT3 WT, suggesting that the STAT3/CDK9 interaction is stabilized in the absence of STAT3 Ser phosphorylation (Figure 4E). Overall, our studies show that CDK8 and CDK9 fine-tune STAT3 nuclear dynamics.

### CDK8 Regulates IL-6-Induced Nuclear Phosphoproteome

**Figure 5:**
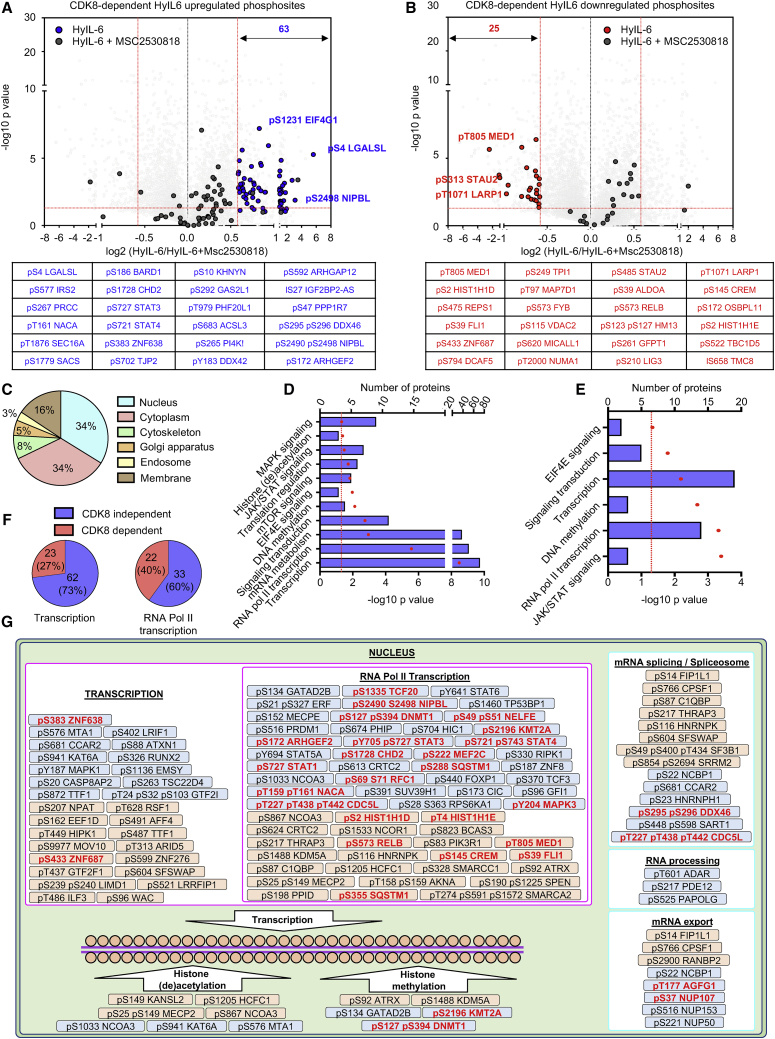
Regulation of the Phosphoproteomic Landscape of IL-6 in Human Primary CD4^+^ Th-1 Cells by CDK8 (A) Volcano plot of the CDK8-dependent HyIL-6-upregulated phosphosites in human primary Th-1 cells (top panel) and the 24 more affected phosphosites (bottom panel). (B) Volcano plot of the CDK8-dependent HyIL-6-downregulated phosphosites in human primary Th-1 cells (top panel) and the 24 more affected phosphosites (lower panel). Phosphopeptides identified in three biological replicates are shown as log-transformed SILAC ratios plotted against log-transformed p values (two-sided t test). Select phosphopeptides are labeled (see Table S2 for full list). For (A) and (B), phosphosites regulated by HyIL-6- in a CDK8-dependent way and changed more than 1.5-fold with a p value of <0.05 are shown in red (decreased) or blue (increased), and highlighted in dark gray is the effect of MSC2530818 on those same phosphosites. (C) GO analysis showing the cellular location of the phosphosites regulated by HyIL-6 in a CDK8-dependent manner. (D) GO analysis showing the main pathways and cellular processes regulated by HyIL-6 in human primary Th-1 cells. (E) GO analysis showing the main pathways and cellular processes regulated by HyIL-6 in human primary Th-1 cells in a CDK8-dependent manner. (F) Pie charts showing the number of HyIL-6-regulated and CDK8-dependent phosphosites involved in the regulation of transcription (left graph) or RNA-Pol-II-mediated transcription (right graph). (G) The scheme shows the cellular location and molecular function of the proteins regulated by phosphorylation in response to HyIL-6 stimulation in human primary CD4^+^ Th-1 cells in a CDK8-dependent fashion as determined by DAVID analysis.

Our data have highlighted a critical role of CDK8 in regulating STAT3 Ser phosphorylation and nuclear dynamics in human Th-1 cells (Figures 3E, 4A, and 4C). Next, we asked which proportion of the IL-6-regulated phosphoproteome was dependent on CDK8 activity. For that investigation, we performed phosphoproteomics studies in Th-1 cells stimulated with HyIL-6 for 15 min in the absence or presence of the CDK8 inhibitor MSC2530818. The combined analysis of our phosphoproteomics study identified 11,035 phosphosites in 3,500 proteins (Figures 5A and 5B; Table S2). To minimize mis-interpretation of the data resulting from off-target effects mediated by the CDK8 inhibitor, we focused our analysis on phosphosites that were induced by HyIL-6 treatment and sensitive to CDK8 inhibition. HyIL-6 treatment induced 162 and repressed 160 phosphosites, of which 88 of them (63 of the upregulated and 25 of the downregulated) were sensitive to CDK8 inhibition (Figures 5A and 5B; Table S2). Consistent with our initial phosphoproteome study (Figure 2), a large fraction (34%) of the phosphoproteomic changes induced by HyIL-6 took place in the nuclei of the cells, based on GO analysis (Figure 5C). GO analysis studies indicated that HyIL-6 stimulation regulated proteins involved in transcription, specifically RNA-Pol-II-mediated transcription and other cellular processes such as histone (de)acetylation and DNA methylation (Figures 5D and 5E). A total of 27% of proteins involved in transcription and 40% of proteins involved in RNA Pol II transcription were affected by CDK8 inhibition (Figure 5F). A schematic view of the nuclear IL-6-induced phosphoproteome and its regulation by CDK8 is presented in Figure 5G. Overall, our data highlight a critical contribution of CDK8 in shaping the IL-6 phosphoproteome by regulating processes associated with RNA-Pol-II-mediated transcription.

### CDK8 Regulates STAT3-Mediated Transcription

**Figure 6:**
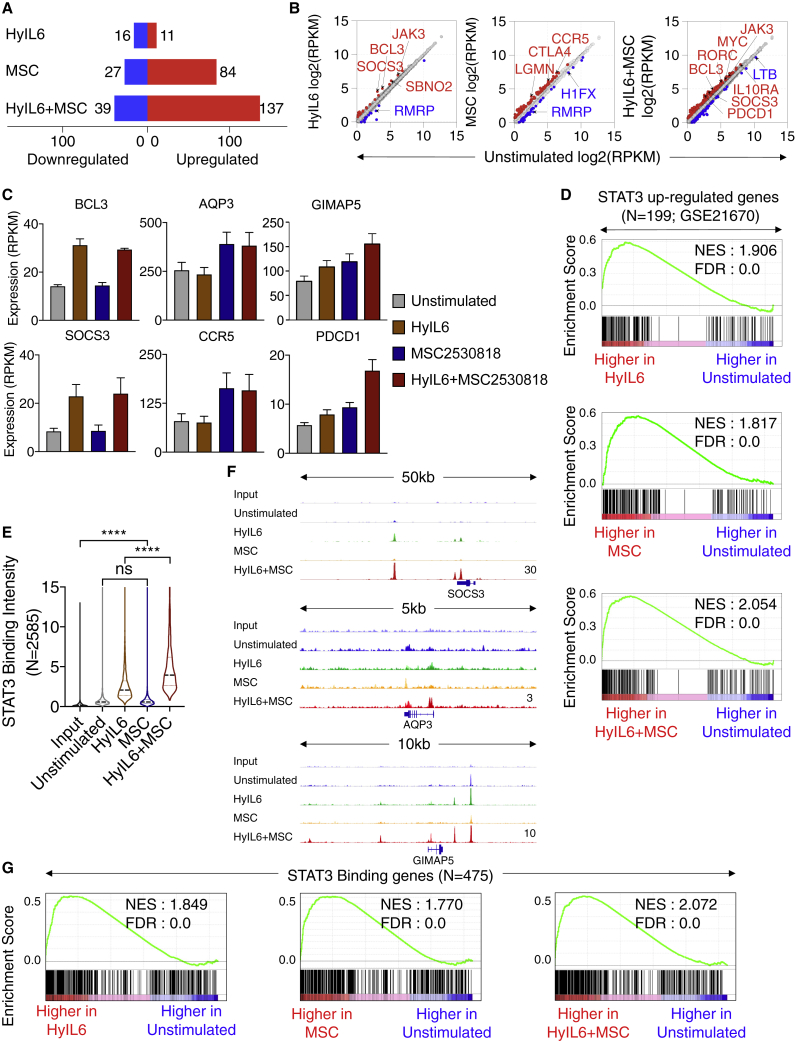
Transcriptional Program Elicited by Interplay between HyIL-6 and CDK8 in Human Primary CD4^+^ Th-1 Cells (A) Number of differentially expressed genes (DEGs; fold chang,e >1.5; p < 0.05) between unstimulated versus HyIL-6-, mesenchymal stem cell (MSC)-, or HyIL-6+MSC-stimulated Th-1 cells in three biological replicates. (B) Scatterplot showing mean gene expression values (n = 3) before (x axis) and after indicated stimulation (y axis). Upregulated (red) and downregulated (blue) genes are highlighted. (C) Representative gene expression across different stimulation. Bars show mean ± SEM. (D) Gene set enrichment analysis (GSEA) (Subramanian et al., 2005) plots for STAT3 upregulated genes (GEO: GSE21670) comparing stimulated versus unstimulated Th-1 transcriptomes. NES, normalized enrichment score; FDR, false discovery rate. (E) Violin plot showing the mean STAT3 binding intensity in n = 2,585 STAT3-bound regions across different stimulations. Peaks are identified by comparing HyIL-6+MSC stimulation and input. The p values were determined by two-tailed Wilcoxon rank-sum test (^∗∗∗∗^p < 0.0001). (F) Representative loci showing STAT3 binding across different stimulations. The height of the tracks are indicated at bottom-right corner of the plots. (G) GSEA plots for 475 STAT3-bound genes comparing stimulated versus unstimulated Th-1 transcriptomes.

**Figure 7:**
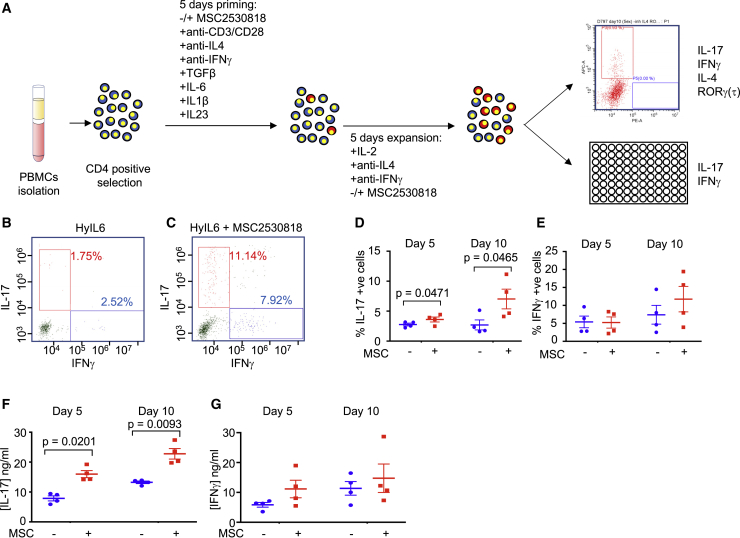
Role of CDK8 Ser727 Phosphorylation of STAT3 in Th-17 Differentiation *In Vitro* (A) Experimental workflow for human Th-17 differentiation *in vitro* from isolated human resting CD4^+^ T cells. (B and C) Dot plot representations of IL-17- and IFNγ-positive cells in populations grown in the presence of HyIL-6 (B) or HyIL-6 + MSC2530818 (C). (D) IL-17-positive cells were identified by flow cytometry in untreated cells or cells treated with 2 μM MSC2530818. Data are percentage of positive cells ± SEM in four biological replicates; p values were calculated using a paired t test. (E) As in (D) but for IFNγ-positive cells. (F) Amount of IL-17 ± SEM in four biological replicates detected in growth media following growth of cells minus or plus inhibitor. (G) Amount of IFNγ ± SEM in four biological replicates detected in growth media following growth of cells minus or plus inhibitor. Statistical significance was calculated by unpaired t test.

We showed that STAT3 nuclear dynamics were regulated by CDK8 activity. Thus, we next asked whether inhibition of CDK8 activity would alter STAT3-dependent gene transcription. For that question, we performed RNA-seq studies in Th-1 cells stimulated with HyIL-6 with or without the CDK8 inhibitor MSC2530818 for 6 h (Figures 6A–6D; Figure S7A). CDK8 inhibition by MSC2530818 neither altered the phosphorylation profile of RBP1 nor blocked transcriptional upregulation induced by HyIL-6 treatment, suggesting that CDK8 inhibition did not result in an overall transcription blockage (Figure 6; Figure S7B) (Czodrowski et al., 2016; Harlen and Churchman, 2017). As previously observed by our laboratory (Martinez-Fabregas et al., 2019), HyIL-6 stimulation alone resulted in changes in the expression pattern of a small number of genes (n = 27) including classical STAT3 targets in Th-1 cells (Figures 6A and 6B, left panel). Treatment with only CDK8 inhibitor led to changes in the expression of 111 genes, of which 84 were upregulated (Figures 6A and 6B, middle panel). The combined HyIL-6 and MSC2530818 treatments exhibited a synergistic effect, leading to changes in the expression of 176 genes (Figures 6A and 6B, right panel), suggesting that CDK8 inhibition induced transcriptional programs by promoting HyIL-6/STAT3-mediated transcription. Differently regulated genes seem to fall into a few categories: genes induced by HyIL-6 treatment, but not regulated by CDK8 inhibition (e.g., BCL3 and SOCS3); genes induced upon CDK8 inhibition but not regulated by HyIL-6 treatment (e.g., AQP3 and CCR5); and genes exhibiting a synergic regulation by HyIL-6/CDK8 inhibition combined treatment (e.g., GIMAP5 and PDCD1) (Figure 6C). Next, we performed, gene set enrichment analysis for evaluating STAT3-mediated transcriptional activity in the absence and presence of CDK8 inhibitor. As expected, genes upregulated by HyIL-6 stimulation were highly enriched in genes known to be upregulated by STAT3 (GEO: GSE21670) (Figure 6D, top panel). Importantly, however, genes upregulated by CDK8 inhibition alone (Figure 6D, middle panel) or in combination with HyIL-6 (Figure 7D, bottom panel) were also highly enriched in known STAT3 targets, indicating that HyIL-6/STAT3 response is mediated by an intrinsic CDK8-dependent axis.

We next asked whether CDK8 could regulate STAT3 binding profiles to chromatin in a genome-wide manner. To assess that question, we carried out STAT3 ChIP-seq in unstimulated or stimulated Th-1 cells with the three conditions described above for 1 h. As expected, in unstimulated Th-1 cells, we detected very low STAT3 DNA binding, which was significantly enhanced upon HyIL-6 treatment (Figure 6E; Figure S7C; Table S3). In Th-1 cells treated with the CDK8 inhibitor alone, we observed levels of STAT3 binding that resembled those obtained in native unstimulated cells (Figure 6E). Strikingly, we observed a synergistic increase in binding intensity of STAT3 across target sites in cells stimulated with the combined HyIL-6/CDK8 inhibitor treatment when compared to HyIL-6 treatment alone, suggesting that inhibition of CDK8 activity amplifies the intensity of HyIL-6-induced STAT3 binding to its target sites (Figure 6E). This increase in STAT3 binding was observed in genes from all three categories described in Figure 6C (Figure 6F). Interestingly, the category of genes not regulated by CDK8 inhibition observed in our RNA-seq study was composed of a set of immediate early genes, such as BCL3 and SOCS3 that are rapidly induced after IL-6 treatment, peaking in the first 2 h of treatment and rapidly declining at later times (Brocke-Heidrich et al., 2006; Starr et al., 1997). It is thus possible that this group of genes is also regulated by CDK8, but our RNA-seq study, which was performed at 6 h of stimulation, has missed the effect. STAT3 binding to DNA was also enhanced when Th-1 cells were treated with the structurally unrelated inhibitor flavopiridol, ruling out that our observations result from off-target effects derived from the use of the MSC2530818 inhibitor (Figures S7D and S7E). As expected, under all conditions tested, STAT3 was binding to a canonical STAT3 GAS sequence motif (Figure S7F). Moreover, genes that were differentially regulated upon stimulation were highly enriched in genes that harbor at least one STAT3-binding site in their promoter and/or enhancer region (Figure 6G), reaffirming a specific effect of CDK8 inhibition on STAT3 binding to the target gene loci and transcriptional activity.

### CDK8 Regulates Th-17 Differentiation

IL-6 regulates inflammatory processes by inducing the differentiation of Th-17 cells. Thus, we asked whether CDK8 activity would modulate Th-17 differentiation. Human primary resting CD4^+^ T cells were isolated from buffy coats and primed for 5 days under Th-17 polarizing conditions (Sekiya and Yoshimura, 2016). Primed cells were further expanded in media containing IL-2, anti-IL-4, and anti-IFNγ in the presence or absence of the CDK8 inhibitor MSC2530818 (Figure 7A). At day 5 or 10 of expansion, cells were analyzed by flow cytometry and ELISA for expression of the indicated cytokines (Figure 7). As previously described, HyIL-6 treatment induced a minor increase in the number of human Th-17 cells, highlighting the challenge of working with these cells *in vitro* (Hakemi et al., 2011; Miyahara et al., 2008). About 2%–3% of the T cells at the end of the polarization protocol were positive for IL-17 (Figures 7B and 7D). CDK8 inhibition led to an average of a 3-fold increase in the number of IL-17-positive cells after 10 days of expansion (Figures 7C and 7D). As expected, we did not see changes in IFNγ expression by the T cells stimulated in the presence of the CDK8 inhibitor (Figures 7B, 7C, and 7E), suggesting a specific regulation of STAT3-mediated Th-17 differentiation by the CDK8 inhibitor. Moreover, CDK8 inhibition resulted in an increase in the levels of secreted IL-17 as measured by ELISA (Figure 7F), but not of IFNγ (Figure 7G). Overall, our data agree with a model where CDK8 modulates STAT3 transcriptional processivity by fine-tuning its gene loci binding dwell-time, leading to a negative regulation of IL-6-mediated activities.

## Discussion

Our study explores how Ser phosphorylation regulates STAT3 activities in human primary T cells. Overall, our study provides molecular evidence that establishes CDK8 as a master regulator of STAT3 transcriptional activities and presents a potential strategy to harness the therapeutic potential of cytokines by fine-tuning CDK8 expression levels and activities in different immune cells.

Although a large body of work in the existing literature support a model in which Ser phosphorylation positively contributes to STAT3 transcriptional activities, our study suggests a negative role of CDK8 activity in STAT3 DNA binding and transcriptional activities in human T cells, mediated at least in part by Ser phosphorylation of STAT3. How can these apparent contradictory observations be reconciled? Previous studies used STAT3 Ser-to-Ala mutants to investigate how Ser phosphorylation regulates STAT3 transcriptional activities (Wakahara et al., 2012; Wen and Darnell, 1997; Wen et al., 1995; Yang et al., 2020). In this context, CDK8 activity remained intact, and therefore, its contribution to STAT3 transcriptional activity was not explored. In agreement with our observations, these studies reported that Ser phosphorylation of STAT3 regulated its chromatin binding dynamics (Wakahara et al., 2012; Yang et al., 2020). STAT3 Ser phosphorylation contributed to destabilization of STAT3 homodimers, resulting in their release from DNA and in STAT3 Tyr dephosphorylation (Yang et al., 2020). However, despite the stronger DNA binding exhibited by the STAT3 S727A mutant, its transcriptional activity was decreased (Wakahara et al., 2012; Wen et al., 1995; Yang et al., 2020). Our study shows that in T cells, CDK8 inhibition prevents STAT3 Ser phosphorylation by IL-6 and increases STAT3 chromatin binding. However, contrary to previous observations, in this context, prolonged STAT3 binding to DNA results in an increased STAT3-dependent transcription. One explanation for these discrepancies in STAT3 activities upon blockage of its Ser phosphorylation could be found in the use of different cell types in the different studies. Although previous studies used cancer cell lines (Wakahara et al., 2012; Wen et al., 1995; Yang et al., 2020), we have used primary human T cells, which thus does not rule out possible differences in the epigenetic landscape between these different cell types that dictates STAT binding profiles. An alternative explanation could be that mutation of S727 in STAT3 and blocking CDK8 activity produce different effects on STAT3 transcriptional activity. Importantly, we show that the STAT3/CDK complex is stabilized in the context of the STAT3 S727A mutant upon IL-6 stimulation. Thus, it is possible that in this context, sustained CDK8 activity, as a result of a stable STAT3/CDK8/DNA complex, could result in transcriptional repression as the one observed for the STAT3 S727A mutant. Our phosphoproteomic study agrees with this model and shows that IL-6 induces the phosphorylation of different transcription factors, including CREM, RelB, and FLI1, in a CDK8-dependent manner, highlighting that active CDK8 elicits a broader regulation of the transcription machinery in response to IL-6 stimulation. Further molecular studies will be required to fully understand how the CDK8/STAT3 interaction ultimately fine-tunes transcriptional output.

IL-6 triggers a strong, albeit transient, phosphorylation of STAT1, but the role that STAT1 phosphorylation plays in fine-tuning IL-6 responses is not clear. STAT1 can form heterodimers with STAT3, having the potential to alter its transcriptional activity (Hirahara et al., 2015). Interestingly, CDK8 phosphorylates both STAT1 and STAT3 (Bancerek et al., 2013), but whether this phosphorylation regulates the formation of STAT1/STAT3 heterodimers is not known at the moment. In addition, STAT1 levels are modulated during inflammation as a consequence of high IFNα/γ expression in this inflammatory environment, leading to altered responses by cytokines triggering classical STAT3 responses, through the modulation of STAT1/3 homo- and hetero-dimers (Ho and Ivashkiv, 2006). Further studies will be required to address whether CDK8 regulates formation of STAT3 homo- and hetero-dimers and if this regulation is altered in inflammatory environments in which STAT levels are different.

The role that CDK8 plays in transcription regulation has been controversial. Although initially CDK8 was identified as a negative regulator of RNA-Pol-II-mediated transcription (Jeronimo and Robert, 2017; Knuesel et al., 2009), more recent studies have described showing that CDK8 can also act as a positive modulator of RNA Pol II transcriptional activities (Chen et al., 2017; Donner et al., 2007, 2010). Our results suggest a negative role of CDK8 in STAT3-mediated transcription. CDK8 activity triggers STAT3 dissociation from chromatin and terminates STAT3-mediated transcription. Supporting our model, a series of recent studies have described that modulation of CDK8 activity, by either small-molecule inhibitors or genetic deletion, fine-tune responses elicited by different immune cells. Specific deletion of CDK8 in NK cells results in an enhancement of their anti-tumor responses (Witalisz-Siepracka et al., 2018), an activity that heavily relies on the action of different cytokines on natural killer (NK) cells (Hu et al., 2019). Moreover, small-molecule inhibitors targeting CDK8 promote the differentiation of T reg cells and Th-17 cells when T cells were placed under T reg polarizing conditions (Akamatsu et al., 2019; Guo et al., 2019) or Th-17 polarizing conditions (Figure 7), respectively. In both instances, the polarizing conditions were enriched in different cytokines, highlighting a potentially broad regulation of cytokine responses by CDK8. Interestingly, CDK8 expression itself is dynamic. Naive T cells express non-detectable levels of CDK8, which are upregulated upon T cell activation and differentiation (Howden et al., 2019). Thus, it is possible that cells can regulate their CDK8 levels to establish different thresholds of cytokine sensitivity. In agreement with this model, despite high levels of STAT3 Tyr phosphorylation and STAT3 chromatin binding induced by IL-6 treatment, we detected very few genes induced by IL-6 in Th-1 cells, which express high levels of CDK8 (Howden et al., 2019).

### Limitations of Study

Our study shows that CDK8 regulates STAT3 transcriptional activities by limiting its binding to the target loci. However, whether this repressor effect results from CDK8-mediated phosphorylation of STAT3 on its S727 residue remains an open question. Previous studies described that STAT3 Ser phosphorylation regulates its chromatin binding dynamics, consistent with our model (Wakahara et al., 2012; Yang et al., 2020). However, comparison of chromatin binding profiles between STAT3 WT and its S727A counterpart would be ideal to further support the relative importance of STAT3 Ser phosphorylation in the regulation of STAT3-DNA binding kinetics and its transcriptional activities. Due to the technical challenges in genetic manipulation of primary human T cells, at present, the genomic tools available to examine the role of STAT3 Ser phosphorylation in regulating its transcriptional activities in human primary T cells are inadequate. Furthermore, elucidating whether CDK8 activity regulates the formation of STAT1/STAT3 homo- and hetero-dimers upon IL-6 stimulation, ultimately impacting IL-6-mediated gene expression programs, remains important.

## STAR★Methods

### Key Resources Table

**Table:** 

REAGENT or RESOURCE	SOURCE	IDENTIFIER
**Antibodies**		

Rat anti-human-CD4-FiTC (Clone A161A1)	Biolegend	Cat#357406; RRID: AB_2562357
Mouse anti-human-CD8-FiTC (Clone SK1)	Biolegend	Cat#344704; RRID: AB_1877178
Mouse anti-human-CD3-BV510 (Clone UCHT1)	Biolegend	Cat#300448; RRID: AB_2563468
Rat anti-human-CD4-PE (Clone A161A1)	Biolegend	Cat#357404; RRID: AB_2562036
Mouse anti-human-CD8-AF700 (Clone HIT8a)	Biolegend	Cat#300920; RRID: AB_528885
Rabbit anti-pSTAT1-Y701-AF647 (Clone 58D6)	Cell Signaling	Cat#8009S; RRID: AB_10860764
Mouse anti-pSTAT1-S727-AF488 (Clone A15158B)	Biolegend	Cat#686410; RRID: AB_2650784
Mouse anti-pSTAT3-Y705-AF488 (Clone 13A3-1)	Biolegend	Cat#651006; RRID: AB_2572084
Mouse anti-pSTAT333-S727-AF647 (Clone A16089B)	Biolegend	Cat#698914; RRID: AB_2750260
Mouse Ultra-LEAF purified anti-human-CD3 (Clone UCHT1)	Biolegend	Cat#300438; RRID: AB_11146991
Mouse anti-pSTAT4-Y693-AF488 (Clone 38/p-Stat4)	BD Biosciences	Cat#558136; RRID: AB_397051
Rabbit anti-pSTAT5-Y694-AF647 (Clone C71E5)	Cell Signaling	Cat#9365S; RRID: AB_1904151
Mouse anti-pSTAT6-Y641-AF488 (Clone 18/P-Stat6)	BD Biosciences	Cat#612600; RRID: AB_399883
Mouse anti-pERK-T202/Y204-AF488 (MILAN8R)	eBiosciences	Cat#53-9109-41; RRID: AB_2574440
Rabbit anti-pAKT-S473-AF488 (Clone D9E)	Cell Signaling	Cat#4071S; RRID: AB_1031106
Rabbit anti-pAKT-T308-AF647 (Clone D25E6)	Cell Signaling	Cat#48646S;RRID: AB_2799341
Rabbit anti-pP90RSK-S380-AF488 (Clone D5D8)	Cell Signaling	Cat#13588S; RRID: AB_2798266
Rabbit anti-pS6R-S240/S244-AF488 (Clone D68F8)	Cell Signaling	Cat#5018S; RRID: AB_10695861
Rabbit anti-pS6R-S235/S236-AF647 (Clone D57.2.2E)	Cell Signaling	Cat#4851S; RRID: AB_10695457
Rabbit anti-pZAP70-Y319/pSYK-Y352-AF647 (Clone 65E4)	Cell Signaling	Cat#82975S; RRID: AB_2800004
Rabbit anti-pCREB-S133-AF488 (clone 87G3)	Cell Signaling	Cat#9187S; RRID: AB_659957
Rabbit anti-pHIS3-S10-AF647	Cell Signaling	Cat#9716S; RRID: AB_330212
Rabbit anti-pGSK3β-S9-AF647 (Clone D85E12)	Cell Signaling	Cat#14332S; RRID: AB_2798453
Rabbit anti-pCFOS-S32-AF647 (Clone D82C12)	Cell Signaling	Cat#8677S; RRID: AB_11178518
Rabbit anti-IRF1-AF647 (Clone D5E4)	Cell Signaling	Cat#14105S; RRID: AB_2798393
Rat anti-IRF4-AF647 (Clone IRF4.3E4)	Biolegend	Cat#646408; RRID: AB_2564048
Mouse anti-IRF7-AF647 (Clone 12G9A36)	Biolegend	Cat#656007; RRID: AB_2563530
Mouse anti-GATA3-AF488 (Clone 16E10A23)	Biolegend	Cat#653807; RRID: AB_2563214
Mouse anti-TBET-AF647 (Clone 4B10)	Biolegend	Cat#644803; RRID: AB_1595573
Mouse anti-HIF1α-AF488 (Clone 546-16)	Biolegend	Cat#359707; RRID: AB_2563975
Rabbit anti-cMYC-AF488 (Clone D84C12)	Cell Signaling	Cat#12855S; RRID: AB_2798045
Mouse anti-O-GlcNAC-AF647 (Clone RL2)	NOVUS Biologicals	Cat#NB300-524AF647; RRID: AB_10001871
Mouse anti-STAT3-APC (Clone M59-50)	BD Biosciences	Cat#560392; AB_1645463
Mouse anti-human-PLCγ1-AF647 (Clone 27/PLC)	BD Biosciences	Cat#557883; RRID: AB_396921
Mouse anti-total-STAT3 (Clone 124H6)	Cell Signaling	Cat#9139S; RRID: AB_331757
Rabbit Anti-total-RPB1 (Clone D8L4Y)	Cell Signaling	Cat#14958S; RRID: AB_2687876
Rabbit anti-pSer2-RPB1 (Clone E1Z3G)	Cell Signaling	Cat#13499S; RRID: AB_2798238
Rabbit anti-pSer5-RPB1 (Clone D9N5I)	Cell Signaling	Cat#13523S; RRID: AB_2798246
Rabbit anti-GAPDH (Clone 14C10)	Cell Signaling	Cat#2118S; RRID: AB_561053
Donkey anti-rabbit-HRP	Stratech	Cat#711-035-152-JIR; RRID: AB_10015282
Donkey anti-mouse-HRP	Stratech	Cat#715-035-150-JIR; RRID: AB_2340770
Rabbit anti-CDK8	Invitrogen	Cat#PA1-21780; RRID: AB_2291488
Rabbit anti-CDK9 (Clone C12F7)	Cell Signaling	Cat#2316S; RRID: AB_2291505
Rat Purified NA/LE anti-human-IL4 (Clone MP4-25D2)	BD Biosciences	Cat#554481; RRID: AB_395421
Mouse Purified NA/LE anti-human-IFNγ (Clone B27)	BD Biosciences	Cat#554698; RRID: AB_395516
Mouse anti-human-IFNγ-AF488 (Clone 4S.B3)	Biolegend	Cat#502517; RRID: AB_493030
Mouse anti-human-IL17A-APC (Clone BL168)	Biolegend	Cat#512334; RRID: AB_2563986
Rabbit anti-CDK8 (Clone G398)	Cell Signaling	Cat#4101S; RRID: AB_1903934

**Biological Samples**		

Human peripheral blood mononuclear cells (Proteomics and p-proteomics studies)	Scottish Blood Transfusion Service	N/A
Human peripheral blood mononuclear cells (ChIP-seq and RNA-seq studies)	StemCell Technologies	Cat#70025

**Chemicals, Peptides, and Recombinant Proteins**		

Recombinant human Interleukin-2	Novartis	Cat#709421
Recombinant human Interleukin-12	Biolegend	Cat#573002
Recombinant human Interleukin-1β	R&D Systems	Cat#201-LB/CF
Recombinant human Interleukin-23	R&D Systems	Cat#1290-IL
Tofacitinib	Stratech	Cat#S2789-SEL
Rapamycin	Stratech	Cat#S1039-SEL
Torin 1	Tocris	Cat#4247
CHIR-99021	Stratech	Cat#G09-901B-SGC
PD184352	Stratech	Cat#S1020-SEL
Roscovitine	Calbiochem	Cat#557360
GDC0941	abCam	Cat#ab141352
PP2	Stratech	Cat#S7008-SEL
VX745	Tocris	Cat#3915
JAK inhibitor VII	Calbiochem	Cat#796041-65-1
AX15836	Tocris	Cat#5843
AZD8055	Stratech	Cat#A8214-APE
KU0063794	Tocris	Cat#3725
KU55933	Stratech	Cat#A4605-APE
KU57788	Stratech	Cat#A8315-SEL
BI-1347	Boehringer Ingelheim	N/A
MSC2530818	Stratech	Cat#S8387-SEL
CDK9 inhibitor II	Calbiochem	Cat#140651-18-9
NVP2	Tocris	Cat#6535
THZ531	Stratech	Cat#A8736-APE
MFH-2-90-1	Kind gift of Dr. Greg Findlay, University of Dundee, UK	N/A
Flavopiridol	Stratech	Cat#S2679-SEL
Recombinant human Hyper-Interleukin-6	Expressed and purified in the lab	N/A
Recombinant human Interleukin-6	Expressed and purified in the lab	N/A

**Deposited Data**		

Phosphoproteomics raw and analyzed data	This paper	ProteomeXchange: PXDX020964
ChIP-seq and RNA-seq data	This paper	GEO: GSE147399

**Experimental Models: Cell Lines**		

Hut78 cells	ATCC	Cat#TIB-161
HEK293T cells	ATCC	Cat#CRL-11268

**Recombinant DNA**		

Human pSEMS-STAT3 WT-meGFP WT	This study	N/A
Human pSEMS-STAT3 S727A-meGFP WT	This study	N/A
Human Hyper-IL6 pAcGP67-A	This study	N/A
Human Interleukin-6 pAcGP67-A	This study	N/A

**Software and Algorithms**		

MaxQuant	Cox and Mann, 2008	https://www.maxquant.org
Andromeda	Cox et al., 2011	https://www.maxquant.org
DAVID GO analysis tools	Huang et al., 2007, 2009	https://david.ncifcrf.gov/home.jsp
FastQC v0.11.8	Babraham Institute Bioinformatics	https://www.bioinformatics.babraham.ac.uk/projects/fastqc/
RSEM v1.3.1	Li and Dewey, 2011	https://deweylab.github.io/RSEM/
edgeR v3.24.0	Robinson et al., 2010	http://bioconductor.org
Datagraph v4.5	Visual Data Tools, Inc	www.visualdatatools.com
PRISM v8.4.0	GraphPad	https://www.graphpad.com/scientific-software/prism/
GSEA v4.0.3	Subramanian et al., 2005	https://www.gsea-msigdb.org/gsea/index.jsp
Metascape	Zhou et al., 2019	https://metascape.org
Bowtie v1.2.2	Langmead et al., 2009	http://bowtie-bio.sourceforge.net/index.shtml
Samtools v1.9	Li et al., 2009	http://www.htslib.org
bamCoverage v3.2.0	Ramírez et al., 2016	https://usegalaxy.eu/
MEME Suite v5.0.2	Bailey et al., 2009	http://meme-suite.org
TOMTOM	Gupta et al., 2007	http://meme-suite.org/tools/tomtom
BEDTools	Quinlan and Hall, 2010	https://bedtools.readthedocs.io/en/latest/
UCSC bigWigAverageOverBed v2	University of California Santa Cruz	https://genome.ucsc.edu
HOMER v4.10	Gupta et al., 2007	http://homer.ucsd.edu/homer/

### Resource Availability

#### Lead Contact

Further information and requests for resources and reagents should be directed to and will be fulfilled by the Lead Contact Ignacio Moraga Gonzalez (imoragagonzalez@dundee.ac.uk).

#### Materials Availability

This study did not generate new unique reagents.

#### Data and Code Availability

The phosphoproteomic data have been deposited in the ProteomeXchange: PXD020964 (www.proteomexchange.com). The raw and processed ChIP-seq and RNA-seq data are deposited to GEO: GSE147399.

### Experimental Model and Subject Details

#### Human Primary T cells

Peripheral blood mononuclear cells (PBMCs) from healthy donors were purified from buffy coats acquired from the Scottish Blood Transfusion Service and used for signaling and phosphoproteomics experiments.

*In vitro* polarized human Th-1 cells generated from human PBMCs acquired from StemCell Technologies (Cat#70025) were used for RNA-seq and ChIP-seq experiments.

#### Cell Lines

Hut78 (cat#TIB-161) and HEK293T (cat#CRL-11268) cells were obtained from ATCC (www.lgcstandards-atcc.org).

### Method Details

#### Protein expression and purification

HyIL-6 (Fischer et al., 1997) cloned into the pAcGP67-A vector (BD Biosciences) in frame with an N-terminal gp67 signal sequence and a C-terminal hexahistine tag was produced using the baculoviral expression system, as previously described (LaPorte et al., 2008). The baculoviral stocks were prepared in *Spodoptera frugiperda* (*Sf9*) cells grown in SF900III media (Invitrogen, #12658027) and used to infect *Trichoplusiani ni* (High Five) cells grown in InsectXpress media (Lonza, #BELN12-730Q) for protein expression. After 48h infection, secreted protein was captured from High Five supernatants using HisPur Ni-NTA resin (Thermo Scientific, #88223) affinity chromatography, concentrated, and purified by size exclusion chromatography on a Enrich SEC 650 1 × 300 column (Biorad), equilibrated in 10 mM HEPES (pH 7.2) containing 150 mM NaCl. Recombinant HyIL-6 was purified to greater than 98% homogeneity.

#### CD4^+^ and CD8^+^ T cell isolation

Peripheral blood mononuclear cells (PBMCs) from healthy donors were purified from buffy coats (Scottish Blood Transfusion Service) by density gradient centrifugation following manufacturer’s instructions (Lymphoprep, StemCell Technologies, #07801). For CD4^+^ and CD8^+^ T cells isolation, 1 × 10^8^ PBMCs per donor were stained with anti-CD4^FiTC^ (Biolegend, #357406) or anti-CD8^FITC^ (Biolegend, #344704) antibodies and isolated by magnetic activated cell sorting (MACS, Miltenyi) using anti-FiTC microbeads (Miltenyi, #130-048-701) according to manufacturer’s instructions to a purity ≥ 99%.

#### Dose-response and kinetic experiments

For dose-response experiments of STAT1 or STAT3 phosphorylation, 96-well plates were prepared with 30 μL of cells at 5 × 10^5^ cells/mL. Cell were then stimulated with different concentrations to obtain the dose-response curves. After stimulation cells were fixed with 2% formaldehyde for 10 minutes at RT.

For kinetics experiments, cell suspensions were stimulated with saturating concentrations of the cytokines (10 nM HyIL-6) as indicated and cells finally fixed with 2% formaldehyde for 10 minutes at RT.

#### Permeabilization, fluorescence barcoding and antibody staining

After fixation, cells were collected by centrifugation at 1200 rpm for 5 min, formaldehyde blocked by washing the cells with 200 μL of PBS containing 5 mg/ml BSA (PBSA) and collected again by centrifugation at 1200 rpm for 5 min. Then, cells were resuspended and permeabilized in ice-cold methanol for 20 minutes on ice. Cells were then fluorescently barcoded (Krutzik and Nolan, 2006) using a combination of different concentrations of amino-acid reactive dyes (PacificBlue #10163, DyLight800 #46421, Thermo Scientific). Finally, cells were pooled and stained with anti-CD3^BV510^ (Biolegend, #300448), anti-CD4^PE^ (Biolegend, #357404), anti-CD8^AF700^ (Biolegend, #300920), anti-pSTAT1-Tyr701^AF647^ (Cell Signaling, #8009S), anti-pSTAT1-Ser727^AF488^ (Biolegend, #686410), anti-pSTAT3-Tyr705^AF488^ (Biolegend, #651006) and anti-pSTAT3-Ser727^AF647^ (Biolegend, #698914). Cells were analyzed in a CytoFlex S flow cytometer (Beckman Coulter) with the individual cell populations being identified by their barcoding pattern and mean fluorescence intensity (MFI) for the different forms of STAT1 or STAT3 measured.

#### Phospho-FLOW

Resting PBMCs isolated as described before from buffy coats or upon activation for three days with ImmunoCult Human CD3/CD28 T Cell Activator (StemCell, #10971) following manufacturer instructions in the presence of 20 ng/mL IL2 (Novartis, #709421) were starved for 24 hours in RPMI 1640 (Invitrogen) containing 10% fetal bovine serum (FBS, Invitrogen, #A3160801) and then stimulated with 10nM HyIL-6 or 0.1 μg/mL anti-CD3 (Biolegend #300438) and 20 ng/mL IL2 (Novartis #709421). Then, cells were fixed with 2% formaldehyde, permeabilized with ice-cold methanol and barcoded as described above. Finally, cells were pooled and stained with anti-CD3^BV510^ (Biolegend, #300448), anti-CD4^PE^ (Biolegend, #357404), anti-CD8^AF700^ (Biolegend, #300920), anti-pSTAT1-Y701^AF647^ (Cell Signaling, #8009S), anti-pSTAT1-S727^AF488^ (Biolegend, #686410), anti-pSTAT3-Y705^AF488^ (Biolegend, #651006) and anti-pSTAT3-S727^AF647^ (Biolegend, #698913), anti-pSTAT4-Y693^AF488^ (BD Biosciences, #558136), anti-pSTAT5-Y694^AF647^ (Cell Signaling, #9365S), anti-pSTAT6-Y641^AF488^ (BD Biosciences, #612600), anti-pERK-T202/Y204^AF488^ (eBiosciences, #53-9109-41), anti-pAKT-S473^AF488^ (Cell Signaling, #4071S), anti-pAKT-T308^AF647^ (Cell Signaling, #48646S), anti-pP90RSK-S380^AF488^ (Cell Signaling, #13588S), anti-pS6R-S240/S244^AF488^ (Cell Signaling, #5018S), anti-pS6R-S235/S236^AF647^ (Cell Signaling, #4851S), anti-pZAP70-Y319/pSYK-Y352^AF647^ (Cell Signaling, #82975S), anti-pCREB-S133^AF488^ (Cell Signaling, #9187S), anti-pHIS3-S10^AF647^ (Cell Signaling, #9716S), anti-pGSK3β-S9^AF647^ (Cell Signaling, #14332S),anti-pCFOS-S32^AF647^ (Cell Signaling, #8677S), anti-IRF1^AF647^ (Cell Signaling, #14105S), anti-IRF4^AF647^ (Biolegend, #646408), anti-IRF7^AF647^ (Biolegend, #656007), anti-GATA3^AF488^ (Biolegend, #653807), anti-TBET^AF647^ (Biolegend, #644803), anti-HIF1α^AF488^ (Biolegend, #359707), anti-MYC^AF488^ (Cell Signaling, #12855S), anti-O-GlcNAC^AF647^ (NOVUS Biologicals, #NB300-524AF647), anti-STAT3^APC^ (BD Biosciences, #560392) and anti-PLCγ1^AF647^ (BD Biosciences, #557883). Cells were analyzed in a CytoFlex S flow cytometer (Beckman Coulter) with the individual cell populations being identified by their barcoding pattern and mean fluorescence intensity (MFI) measured.

#### Phosphoproteomics

Resting CD4^+^ T cells were labeled with anti-CD4-FiTC antibody (Biolegend, #357406) and isolated from human PBMCs by magnetic activated cell sorting (MACS, Miltenyi) using anti-FiTC microbeads (Miltenyi, Cat#130-048-701) following manufacturer instructions. Subsequently, resting CD4^+^ T cells were activated under Th-1 polarizing conditions. Briefly, 3x10^7^ resting human CD4^+^ T cells per donor were primed for three days with ImmunoCult Human CD3/CD28 T Cell Activator (StemCell, #10971) following manufacturer instructions in the presence of 20 ng/mL IL2 (Novartis #709421), 20 ng/mL IL12 (BioLegend, #573002) and 10 ng/mL anti-IL4 (BD Biosciences, #554481). Then, cells were split into three different conditions light SILAC media (40 mg/mL L-Lysine K0 (Sigma, #L8662) and 84mg/mL L-Arginine R0 (Sigma, #A8094)), medium SILAC media (49 mg/mL L-Lysine U-13C6 K6 (CKGAS, #CLM-2247-0.25) and 103 mg/mL L-Arginine U-13C6 R6 (CKGAS, #CLM-2265-0.25)) and heavy SILAC media (49.7 mg/mL L-Lysine U-13C6,U-15N2 K8 (CKGAS, #CNLM-291-H-0.25) and 105.8 mg/mL L-Arginine U-13C6,U-15N2 R10 (CKGAS, #CNLM-539-H-0.25)) prepared in RPMI SILAC media (Thermo Scientific, #88365) supplemented with 10% dialyzed FBS (HyClone, #SH30079.03), 5 mL L-Glutamine (Invitrogen, #25030024), 5 mL Pen/Strep (Invitrogen, #15140122), 5 mL MEM vitamin solution (Thermo Scientific, #11120052), 5 mL Selenium-Transferrin-Insulin (Thermo Scientific, #41400045) and expanded in the presence of 20 ng/mL IL2 and 10 ng/ml anti-IL4 for another 10 days in order to achieve complete labeling. Incorporation of medium and heavy version of Lysine and Arginine was checked by mass spectrometry and samples with an incorporation greater than 95% were used. After expansion, cells were starved without IL2 for 24 hours before stimulation with 10 nM HyIL-6 in the presence or absence of 2 μM MSC2530818 (Stratech, # S8387-SEL) for 15 minutes. Cells were then washed three times in ice-cold PBS, mix in a 1:1:1 ratio, resuspended in SDS-containing lysis buffer (1% SDS in 100mM Triethylammonium Bicarbonate buffer (TEAB)) and incubated on ice for 10 minutes to ensure cell lysis. Then, cell lysates were centrifuged at 20000 g for 10 minutes at +4°C and supernatant was transferred to a clean tube. Protein concentration was determined by using BCA Protein Assay Kit (Thermo, #23227), and 10 mg of protein per experiment were reduced with 10mM dithiothreitol (DTT, Sigma, #D0632) for 1 hour at 55°C and alkylated with 20mM iodoacetamide (IAA, Sigma, #I6125) for 30 min at RT. Protein was then precipitated using six volumes of chilled (−20°C) acetone overnight. After precipitation, protein pellet was resuspended in 1mL of 100mM TEAB and digested with Trypsin (1:100 w/w, Thermo, #90058) and digested overnight at 37°C. Then, samples were cleared by centrifugation at 20000 g for 30 min at +4°C, and peptide concentration was quantified with Quantitative Colorimetric Peptide Assay (Thermo, #23275).

Digested samples were fractionated to reduce sample complexity and increase the efficiency of phosphopeptide enrichment. Briefly, peptides (approx. 3.5 mg per sample) were resuspended in 200uL Buffer A (10mM ammonium formate), separated on a XBridge Peptide BEH column (Waters, C18, 3.5 μM, 4.6 × 250 mm) after initially trapped on a XBridge trap cartridge (Waters, C18, 3.5 μM, 4.6 × 20 mm) using an Ultimate 3000 RSLCnano system (Thermo Scientific). Peptides were resolved using a gradient (102 min, 0.8 ml/min) of Buffer A (10mM ammonium formate) and Buffer B (10mM ammonium formate, 90% acetonitrile): 8% Buffer B for 6 min, 8%–45% Buffer B for 54 min, 45%–100% Buffer B for 5 min, 100% Buffer B for for 16 min and 100%–2% Buffer B for 21 min. 80 Fractions were collected using a WPS-3000FC autosampler (Thermo Scientific) of 1 minute (0.8 ml) duration from 1-80 min over the chromatogram. These fractions were then concatenated to 20 fractions to provide a similar quantity of peptide per fraction based on the online (U3000 Variable Wavelength Detector) (Thermo Scientific) UV values of the eluted peptides at 220 nm. These concatenated fractions were taken to dryness (EZ-2 Plus centrifugal evaporator, Genevac) prior to suspending for nLC-MS analysis.

Phosphopeptide enrichment in the peptide fractions generated as described above was carried out using MagResyn Ti-IMAC following manufacturer instructions (2BScientific, MR-TIM002). Phosphopeptide samples were analyzed using a nanoflow liquid chromatography system (Ultimate 3000 RSLCnano system, Thermo Scientific) coupled to a Q Exactive Plus Mass Spectrometer (Thermo Scientific). Samples (10 μl) were loaded onto a C18 trap column and washed for 5 minutes with 0.1% formic acid. Peptides were resolved using a gradient (170 min, 0.3 μl/min) of buffer A (0.1% formic acid) and buffer B (80% acetonitrile in 0.08% formic acid): 5% buffer B for 5 min, 5%–35% buffer B for 125 min, 35%–98% buffer B for 2 min, 98% buffer B for 20 min, 98%–2% buffer B for 1 min and 2% buffer B for 17 min. Peptides, initially trapped on an Acclaim PepMap 100 C18 colum (100 μm x 2 cm, Thermo Scientific), were separated on an Easy-Spray PepMap RSLC C18 column (75 μm x 50 cm, Thermo Scientific), and finally transferred to a Q Exactive Plus Mass Spectrometer via an Easy-Spray source with temperature set at 50°C and a source voltage of 2.0kV. For the identification of peptides, a top 15 method (1 MS plus 15 MS^2^, 150 min acquisition) consisting of full scans and mass range (m/z) between 350 to 1600 (m/z) for MS search and 200 to 2000 (m/z) for MS^2^ search was used. For the MS scan the Q Exactive Plus Mass Spectrometer was operated in a data dependent acquisition mode, resolution of 70,000 with a lock mass set at 445.120024 and max fill time of 20 ms. For the MS^2^ scan Q Exactive Plus Mass Spectrometer was operated in a centroid mode, resolution of 15,000 with isolation window = 1.4 (m/z), normalized collision energy = 27, max fill time of 100 ms and dynamic exclusion of 45.0 s.

#### Inhibition of Ser727 STAT3 phosphorylation

Resting CD4^+^ T cells were labeled with anti-CD4-FiTC antibody (Biolegend, #357406) and isolated from human PBMCs by magnetic activated cell sorting (MACS, Miltenyi) using anti-FiTC microbeads (Miltenyi, Cat#130-048-701) following manufacturer instructions. Then, CD4^+^ T cells were activated for three days with ImmunoCult Human CD3/CD28 T Cell Activator (StemCell, #10971) following manufacturer instructions in the presence of 20 ng/mL IL2 (Novartis, #709421). After activation, cells were expanded for 5 days in the presence of 20ng/mL IL2. Then, cells were starved of IL2 for 24 hours before stimulation with 10nM HyIL-6 in the presence or absence of different inhibitors [2 μM Tofacitinib (Stratech, #S2789-SEL), 2 μM Rapamycin (Stratech, #S1039-SEL), 2 μM Torin1 (Tocris, #4247), 2 μM CHIR-99021 (Stratech, #G09-901B-SGC), 2 μM PD184352 (Stratech, #S1020-SEL), 2 μM Roscovitine (Calbiochem, #557360), 2 μM GDC0941 (abCam, #ab141352), 2 μM PP2 (Stratech, #S7008-SEL), 2 μM VX745 (Tocris, #3915), 2 μM JAK inhibitor VII (Calbiochem, #796041-65-1), 2 μM AX15836 (Tocris, #5843), 2 μM AZD8055 (Stratech, #A8214-APE), 2 μM KU0063794 (Tocris, #3725), 2 μM KU55933 (Stratech, #A4605-APE), 2 μM KU57788 (Stratech, #A8315-APE), 2 μM BI-1347 (a kind gift of Boehringer Ingelheim), 2uM MSC2530818 (Stratech, #S8387-SEL), 2 μM CDK9 inhibitor II (Calbiochem, #140651-18-9), 2 μM NVP2 (Tocris, #6535), 2 μM THZ531 (Stratech, #A8736-APE), 2 μM MFH-2-90-1 (a kind gift of Dr. Greg Findlay, University of Dundee, UK) and 2 μM Flavopiridol (Stratech, #S2679-SEL)] as indicated.

#### Western blotting

Cells were rinsed in ice-cold PBS then lyzed in RIPA buffer (Thermo Scientific) supplemented with protease inhibitor cocktail (ROCHE), 5 mM sodium fluoride, 2 mM sodium orthovanadate and 0.2 mM PMSF incubating on ice for 15 min. Lysates were cleared by centrifugation at 20,000 g for 15 min at 4°C then protein concentrations determined using Coomassie Protein Assay Kit (Thermo Scientific, UK). For each sample, 30 μg of total protein were separated on 4%–12% Bis-Tris polyacrylamide gels (NuPAGE, Invitroge) in MES SDS running buffer then blotted onto Protran 0.2 mM Nitrocellulose (GE Healthcare, UK). Membranes were probed with 1:1000 dilution of the appropriate primary antibody anti-total-STAT3 (Cell Signaling, #9139S), anti-total-RPB1 (Cell Signaling, #14958), anti-pSer2-RPB1 (CellSignaling, #13499), anti-pSer5-RPB1 (CellSignaling, #13523) and anti-GAPDH (Cell Signaling, #2118S). 1:5000 dilution of donkey anti-rabbit-HRP (Stratech, 711-035-152-JIR) or donkey anti-mouse-HRP (Stratech, 715-035-150-JIR) as the secondary antibody. Immobilon Western Chemiluminescent HRP substrate (Millipore, UK) was used for visualization.

#### Proximity ligation assay

Resting CD4^+^ T cells were labeled with anti-CD4-FiTC antibody (Biolegend, #357406) and isolated from human PBMCs by magnetic activated cell sorting (MACS, Miltenyi) using anti-FiTC microbeads (Miltenyi, Cat#130-048-701) following manufacturer instructions. Then, CD4^+^ T cells were activated for three days with ImmunoCult Human CD3/CD28 T Cell Activator (StemCell, #10971) following manufacturer instructions in the presence of 20 ng/mL IL2 (Novartis, #709421). After activation, cells were expanded for 5 days in the presence of 20ng/mL IL2. Then, cells were starved of IL2 for 24 hours before stimulation as indicated and 10^5^ cells were used per experiment. Cells were attached to coverslips by incubating them at 37°C for 1 hour in PBS, then PBS was replaced with RPMI supplemented with 10% FBS and cells stimulated as described. After stimulation, cells were fixed with 2% formaldehyde for 10 minutes at RT, permeabilized with ice-cold methanol for 20 minutes on ice and stained with anti-STAT3 (Cell Signaling, #9139S) and anti-CDK8 (Invitrogen, #PA1-21780) or anti-STAT3 (Cell Signaling, #9139S) and anti-CDK9 (Cell Signaling, #2316S) for Proximity Ligation Assays following manufacturer instructions (Sigma, #DUO92008).

#### Chromatin immunoprecipitation by sequencing (ChIP-Seq)

*In vitro* polarized human Th-1 cells generated from human PBMCs (StemCell Technologies, Cat#70025) were expanded in the presence of IL-2 for 10 days and cells were then washed with complete media and rested for 24 hr starvation in the absence of IL-2, these cells were then either not-stimulated (control) or stimulated with IL-6 or different IL-6 variants for 1 hr, cells were then immediately fixed with 1% methanol-free formaldehyde (Formaldehyde 16%, Methanol-Free, Fisher Scientific, PA, USA) at room temperature for 10mn with gentle rocking cells were then washed twice with cold PBS. For each STAT3 ChIP-seq library sample, approximately 10 × 106 cells were used and the fixed cell palettes were kept at −80°C prior to further processing. The ChIPseq experiments were performed as previously described (Martinez-Fabregas et al., 2019) with some modification as described below. In brief, the frozen cell pellets were thawed on ice and washed once with 1 mL cold PBS by centrifugation at 5000 RPM for 5 min, the resulting cell pellets were re-suspended in 500 uL of lysis buffer (1X PBS, 0,5% Triton X-100, cOmplete EDTA-free protease inhibitor cocktail, Roche Diagnostics, Basel, Switzerland) and incubated for 10 min on ice, followed by a 5 min centrifugation at 5000 RPM. Then the pellets were washed once with 1 mL of sonication buffer (1X TE, 1: 100 protease inhibitor cocktail), re-suspended in 750 uL of sonication buffer (1X TE, 1: 100 protease inhibitor cocktail and 0,5 mM PMSF) and sonicated for 20 cycles (on-20sec and off-45sec) on ice using VCX-750 Vibra Cell Ultra Sonic Processor (Sonics, USA). The sonicated lysates were centrifuged 20 min at 14000 RPM and the clear lysate supernatants were collected and incubated with 30 uL of Protein-A Dynabeads (ThermoFisher, USA) that were pre-incubated with incubated with 10 ug of anti-STAT3 antibody (anti-Stat3, 12640S, Cell Signaling Technology) at 4°C overnight with gentle rotation. Next day, the beads were washed 2 times with RIPA-140 buffer (0.1% SDS, 1% Triton X-100, 1 mM EDTA, 10 mM Tris pH 8.0, 300 mM NaCl, 0.1% NaDOC), 2 times with RIPA-300 buffer (0.1% SDS, 1% Triton X-100, 1 mM EDTA, 10 mM Tris, 300 mM NaCl, 0.1% NaDOC), 2 times with LiCl buffer (0.25 mM LiCl, 0.5% NP-40, 1 mM EDTA, 10 mM Tris pH 8.0, 0.5% NaDOC), once with TE-0,2% Triton X-100 and once with TE buffer. Crosslinks were reversed by incubating the bound complexes in 60 uL TE containing 4.5 uL of 10% SDS and 7.5 uL of 20 mg/mL of proteinase K (Thermofisher, USA) at 65°C overnight for input samples, we used 6 uL of 10% SDS and 10 μL of 20 mg/mL of proteinase K. Then, the supernatants were collected using a magnet and beads were further washed one in TE 0.5M NaCl buffer. Both supernatants were combined, and DNA was extracted with phenol/chloroform, followed by precipitation with ethanol and re-suspended in TE buffer. The library was constructed following the manufacturer protocol of the KAPA LTP Library Preparation Kit (KAPA Biosystems, Roche, Switzerland). ChIP DNA libraries were ligated with the Bioo scientific barcoded adaptors (BIOO Scientific, Perkin Elmer, USA) with T4 DNA ligase according to KAPA LTP library preparation protocol and the ligated ChIP DNA libraries were purified with 1.8x vol. Agencourt AMPure XP beads and PCR amplified using KAPA hot start High-Fidelity 2X PCR Master Mix and NextFlex index primers (Bioo Scientific, PerkinElmer) for 12 cycle by following thermocycler cycles: 30 s hot start at at 98°C, followed by 12 cycle amplification [98°C for 10 s, 60°C for 30 s and 72°C for 30 s] and final extension at 72°C for 1 min. The amplification and quality of the ChIPseq libraries were checked by running 10% of the samples in E-Gel Agarose Gels with SYBR Safe DNA Gel Stain (ThermoFisher Scientific, USA), and if necessary, samples were reamplified additional four cycles using the same thermocycler protocol described above. Then, the libraries were purified and size-selected using Agencourt AMPure XP beads (1.25x vol. to remove short fragments. The concentration of ChIP-DNA libraries was measured by Qubit-4 fluorometer (ThermoFisher, USA) and equal amounts of each sample were pooled and 50 bp paired-end reads were sequenced on an Illumina 4000 platform by GENEWIZ technology (GENEWIZ, USA).

#### RNA-sequencing

For RNA-seq library preparation, *in vitro* polarized human Th-1 cells generated from human PBMCs (StemCell Technologies, Cat#70025) either not stimulated or stimulated with HyIL-6 in the presence or absence of 2 μM MSC2530818 variants at 37°C for 6 hr, total RNA was extracted and RNaseq libraries were prepared by Edinburg Sequencing Core facility.

#### CDK8 and CDK9 knock-down in HEK293T cells

CDK8 and CDK9 knock-down in HEK23T cells was done using Lipofectamine RNAiMAX following manufacturer instructions. Briefly, cell were seeded at 6x10^5^ per well in 6-well plates and transfected with siRNA SMARTPools against CDK8 (Dharmacon, Cat# LQ-003242-00-0005) and/or CDK9 (Dharmacon, Cat# LQ-003242-00-0005) 24 hours later. Cells were cultured for 48 hours in the presence of the siRNA mixture before using them for FACS or western blot analysis. Knock-down efficiency was checked by western blotting using anti-CDK8 (G398, Cat# 4101), anti-CDK9 (C12F7, Cat# 2316) and anti-GAPDH (14C10, Cat# 2118).

#### *In vitro* CDK assay

*In vitro* CDKs phosphorylation reactions (20 μL total volume) were as follows: 10 μL of 2x CDK phospho assay buffer (50mM β-glycerophosphate pH 7.4, 10mM MgCl_2_, 10mM NaF, 1mM DTT) with 100 μM ATP and 5 μL of STAT3 at 20ng/μL. Reactions were initiated by adding 5 μL of varying amounts of CDK7, CDK8 or CDK9. Reactions were incubated for 30 minutes at 30°C and stopped by adding 5 μL of 4x SDS sample buffer and heating to 95°C for 5 minutes. All kinase reactions were performed at least three times. ATP was purchased from Sigma (Cat# A2383-10G), human recombinant CDKs were purchased from Thermo (CDK7/CyclinH/MNAT1 Cat# PV3868, CDK8/CyclinC Cat# PV4402 and CDK9/CyclinK Cat# PV4335) and human recombinant STAT3 was purchased from NovusBiologicals (Cat# H0000677-P01-10 μg).

#### Crispr/CAS9 generation of STAT3 KnD Hut78 cells

5 μL of 200 μM Alt-R CRISPR -Cas9 crRNA (IDT, Hs.Cas9.STAT3.1.AF) were added to 5 μL of 200 μM Alt-R CRISPR-Cas9 tracrRNA (IDT, #1072532), heated to 95°C for 5 min and finally the tube cooled to room temperature. Then, 1.2 μL RNA duplex was mixed with 1.7 μL Alt-R S.p. HiFi Cas9 Nuclease V3 (IDT, #1081059) and 2.1 μL sterile PBS and incubated at RT for 20 min. 2 × 105 Hut78 cells resuspended in 8 μL of buffer R (Neon Transfection System Kit, Thermo) were added to the tubes with the RNP complexes. The electroporation parameters used were: three pulses of 1,325 V with a pulse width of 10 ms. Then, reactions were added directly into antibiotic-free media in a well of the 96-well plate and incubated at 37°C for 16 hr. HeLa cells electroporated with RNP particles were transferred into IMDM media containing 10% FCS and Pen/Strep, expanded and finally individual clones were isolated and tested for STAT3 expression levels.

#### STAT3 KnD Hut78 Cells S727A STAT3 Reconstitution

STAT3 KnD Hut78 cells were reconstituted with a plasmid coding for STAT3 WT or STAT3 S727A mutants by electroporation. 2 × 107 cells were resuspended in 0.25 mL of Ingenio electroporation solution (Mirus, #MIR50111), 30 μg of the appropriate construct added and the mix transferred into 4 mm gap-width cuvettes and incubated at RT for 15 minutes. Cells were electroporated using the BioRad X-Gene Pulser system (0.28 kV, 960 μF), cells transferred to pre-warmed media without Pen/Strep and allow to recover for 24 hours.

#### T cells population differentiation

Resting CD4^+^ T cells isolated as described above were activated under Th-1 or Th-17 polarizing conditions. Briefly, resting human CD4^+^ T cells freshly isolated were activated using ImmunoCult Human CD3/CD28 T Cell Activator (StemCell, Cat#10971) following manufacturer instructions for 3 days in the presence of the cytokines required for the different CD4^+^ T cells populations: Th-1 (IL-2 (20 ng/ml), anti-IL-4 (10 ng/ml, BD Biosciences, Cat#554481), IL-12 (20 ng/ml)) or Th-17 (IL-1β (10 ng/ml, R and D Systems, Cat#201-LB/CF), IL-23 (10 ng/ml, R and D Systems, Cat#1290-IL), anti-IL-4 (10 ng/ml, BD Biosciences, Cat#554481), anti-IFNγ (10 ng/ml, BD Biosciences, Cat#554698)). After three days of priming, cells were expanded in the presence of IL-2 (20 ng/ml). Th-1 and Th-17 cells were restimulated for 6 hr in the presence of PMA (100 ng/ml, Sigma, Cat#P8139), Ionomycin (1 μM, Sigma, I0634) and Brefeldin A (5 μg/ml, Sigma, B7651) before FACS analysis. In all cases cells were fixed with 2% formaldehyde and prepared to be analyzed by FACS. Cells were then permeabilised with Saponin 2% in PBS for 20 min at room temperature and then stained in Saponin 2% in PBS with the appropriate antibodies: Th-1 ((anti-CD3^BV510^ (1:100, Biolegend, Cat#300448), anti-CD4^PE^ (1:100, Biolegend, Cat#357404), anti-CD8^AF700^ (1:100, Biolegend, Cat#300920), anti-IFNγ^AF488^ (1:100, Biolegend, Cat#502517)) and Th-17 ((anti-CD3^BV510^, anti-CD4^PE^, anti-CD8^AF700^, anti-IL17A^APC^ (1:100, Biolegend, Cat#512334)) and analyzed in a CytoFLEX S (Beckman Coulter).

### Quantification and Statistical Analysis

#### Bioinformatics

The following software were used:

MaxQuant (Cox and Mann, 2008)
Andromeda (Cox et al., 2011)
DAVID GO analysis tool (Huang et al., 2007, 2009)
FastQC v0.11.8 (www.bioinformatics.babraham.ac.uk)
RSEM v1.3.1 (Li and Dewey, 2011)
edgeR v3.24.0 (Robinson et al., 2010)
Datagraph v4.5 (www.visualdatatools.com)
PRISM v8.4.0 (https://www.graphpad.com/scientific-software/prism/)
GSEA v4.0.3 (Subramanian et al., 2005)
Metascape (Zhou et al., 2019)
Bowtie v1.2.2 (Langmead et al., 2009)
Samtools v1.9 (Li et al., 2009)
bamCoverage v3.2.0 (Ramírez et al., 2016)
MEME Suite v5.0.2 (Bailey et al., 2009)
TOMTOM (Gupta et al., 2007)
BEDTools (Quinlan and Hall, 2010)
UCSC bigWigAverageOverBed v2 (https://genome.ucsc.edu)
HOMER v4.10 (Gupta et al., 2007)

#### Mass spectrometry data analysis

Q Exactive Plus Mass Spectrometer .RAW files were analyzed, and peptides and proteins quantified using MaxQuant (Cox and Mann, 2008), using the built-in search engine Andromeda (Cox et al., 2011). All settings were set as default, except for the minimal peptide length of 5, and Andromeda search engine was configured for the UniProt *Homo sapiens* protein database (release date: 2018_09). Peptide and protein ratios only quantified in at least two out of the three replicates were considered, and the p values were determined by Students t test and corrected for multiple testing using the Benjamini–Hochberg procedure (Benjamini and Hochberg, 1995).

### DAVID GO analysis tool (Huang et al., 2007, 2009) was used to find statistically over-represented gene ontology (GO) categories in the proteomic data

#### RNA-Seq analysis

The quality of libraries was inspected by FastQC v0.11.8. The expression level of mRNA in each library was quantified by ‘rsem-calculate-expression’ in RSEM v1.3.1 (Li and Dewey, 2011) using default parameters except ‘–bowtie-n 1–bowtie-m 100–seed-length 28–paired-end’. The bowtie index required by RSEM software was generated by ‘rsem-prepare-reference’ on all RefSeq genes, downloaded from UCSC table browser on April 2017. edgeR v3.24.0 (Robinson et al., 2010) package was used to normalize gene expression among all libraries and identify differentially expressed genes among samples with following constraints: fold change ≥ 1.5, p value ≤ 0.05. Scatter and bar plots were drawn by Datagraph v4.5 and PRISM v8.4.0, respectively. Geneset enrichment analysis was performed by GSEA v4.0.3 (Subramanian et al., 2005) with default parameters except ‘-collapse No_Collapse -permute gene_set’. Pathway analysis of differentially expressed genes was performed by Metascape (Zhou et al., 2019) on all GO terms related to biological processes, KEGG Pathways, BioCarta Gene Sets and Hallmark Gene Sets.

#### ChIP-Seq analysis

The quality of libraries was inspected using FastQC v0.11.8. All sequencing reads were aligned to human reference genome (GRCh37; hg19) using bowtie v1.2.2 (Langmead et al., 2009) with default pair-end alignment settings and additional parameters ‘–chunkmbs 1000 S -m 1’. The index for reference genome was constructed by using ‘bowtie-build’ with default parameters. Sorting and indexing of the aligned reads were conducted by Samtools v1.9 (Li et al., 2009). The genome-wide binding profiles (i.e., bigWig files) were generated by bamCoverage v3.2.0 (Ramírez et al., 2016) using parameters ‘–normalizeUsing BPM–minMappingQuality 30–ignoreDuplicates–extendReads 250–blackListFileName hg19.blacklist.bed’. The binding profiles were visualized using IGV genome browser v2.7.0 (Robinson et al., 2011). Binding peaks were called by ‘callpeaks’ procedure from MACS2 v2.1.2 (Zhang et al., 2008) using default parameters except ‘-f BAMPE–nomodel -t treatment -c input’. The identified peaks were further screened against ‘hg19 blacklisted’ genomic regions, mitochondrial DNA, and pseudo-chromosomes. *De novo* motif findings were performed in 200 bp surrounding the summit of n = 500 top bound regions using MEME Suite v5.0.2 (Bailey et al., 2009) with default parameters except ‘-maxsize 10000000 -dna -mod zoops -nmotifs 10’. *De novo* motifs were compared against all JASPAR known motifs by TOMTOM (Gupta et al., 2007). STAT3 shared bound regions in HyIL6 (n = 540) or HyIL-6+MSC (n = 2585) stimulated cells were generated by the intersection between bound regions from n = 3 donor using BEDTools (Quinlan and Hall, 2010). STAT3 binding intensity in shared bound regions was calculated by UCSC bigWigAverageOverBed v2 with default parameters and the mean signal intensity was visualized by PRISM v8.4.0. The shared STAT3 bound regions were annotated with the nearest gene by ‘annotatePeaks’ from HOMER v4.10 (Gupta et al., 2007), yielding 475 unique genes. Statistical analyses were performed using the Two-tailed parametric and non-parametric tests as appropriate.

#### Statistical analysis

Statistical signinficance of differential induction of phophopeptide ratios only quantified in at least two out of the three replicates were considered, and the p values were determined by Students t test and corrected for multiple testing using the Benjamini–Hochberg procedure (Benjamini and Hochberg, 1995). For RNA-seq, statistical significance was calculated by edgeR v3.24.0 (Robinson et al., 2010) package. Calculation of statistical significance for binding intensity of STAT3 ChIP-seq was conducted by non-parametric two-tailed Wilcoxon test. Further information related to the statistical analyses performed is provided in the figure legends.

## Supplemental Information

Document S1. Figures S1–S7

Table S1. HyIL-6-Induced Changes in the Phosphoproteome of Primary Human CD4+ Th1 Cells, Related to Figures 2, S3, and S4

Table S2. Effect of CDK8 Inhibition in the Phosphoproteomics Changes Induced by HyIL-6 in Primary Human CD4+ Th1 Cells, Related to Figure 5

Table S3. Differentially Expressed and/or STAT3-Bound Genes in Response to HyIL6, MSC, or HyIL6+MSC Stimulation, Related to Figures 6 and S7

Document S2. Article plus Supplemental Information
