# Supplementary material for: CDK8 Fine-Tunes IL-6 Transcriptional Activities by Limiting STAT3 Resident Time at the Gene Loci
Source: Cell Rep. 2020 Dec 22;33(12):108545. doi: 10.1016/j.celrep.2020.108545 (PMC7773550; doi:10.1016/j.celrep.2020.108545)
Supplement: Document S1. Figures S1–S7 [file mmc1.pdf]

Cell Reports, Volume *33*

Supplemental Information

CDK8 Fine-Tunes IL-6 Transcriptional Activities by Limiting STAT3 Resident Time at the Gene Loci

Jonathan Martinez-Fabregas, Luopin Wang, Elizabeth Pohler, Adeline Cozzani, Stephan Wilmes, Majid Kazemian, Suman Mitra, and Ignacio Moraga

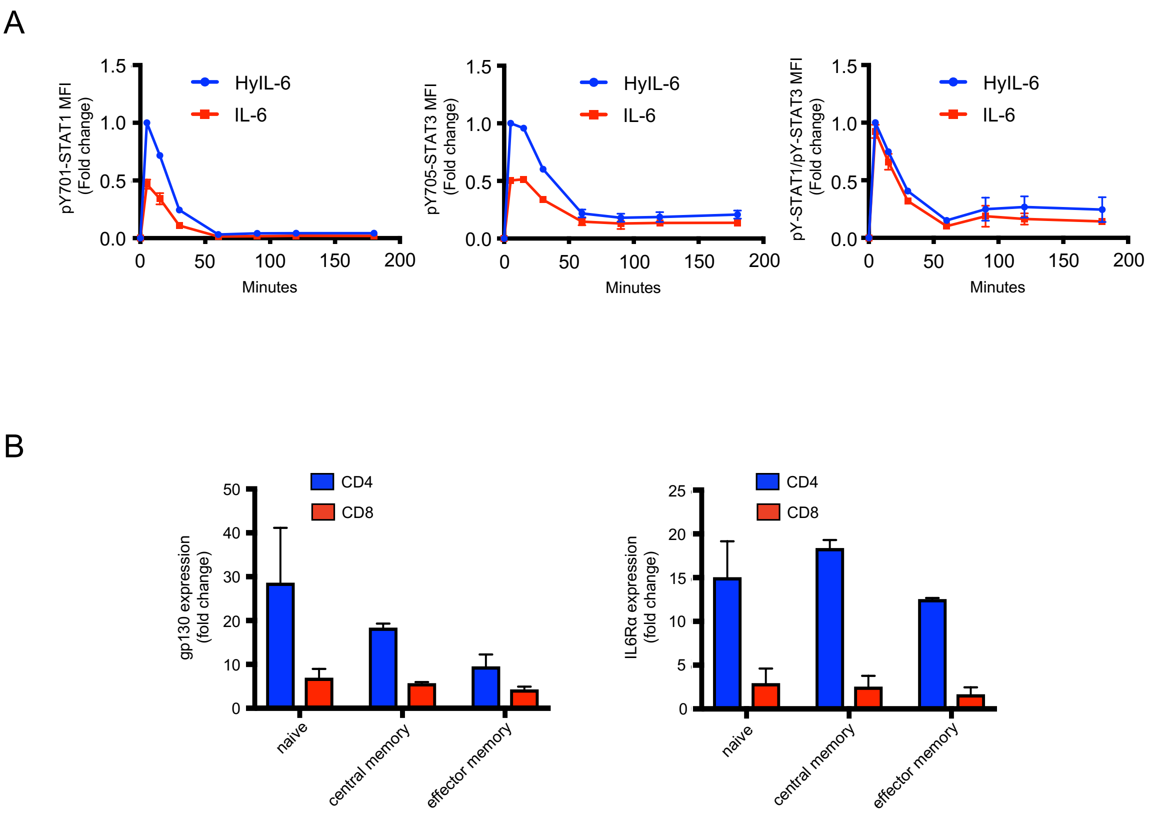

Supplemental Figure S1. HyIL-6 vs IL-6 effect on human primary activated CD4+ T cells and expression levels of gp130 and IL-6Rα in different subpopulations of human T cells. Related to Figure 1. A) STAT1 and STAT3 phosphorylation in response to exposure time of HyIL-6 and IL-6 stimulation in activated primary human CD4^{+} T cells. **B) L**evels of expression of gp130 (left panel) and IL-6Rα (right panel) expressed as fold change in different population of resting primary human CD4^{+} and CD8^{+} T cells. Data represents mean ± SEM calculated from three individual biological replicas.

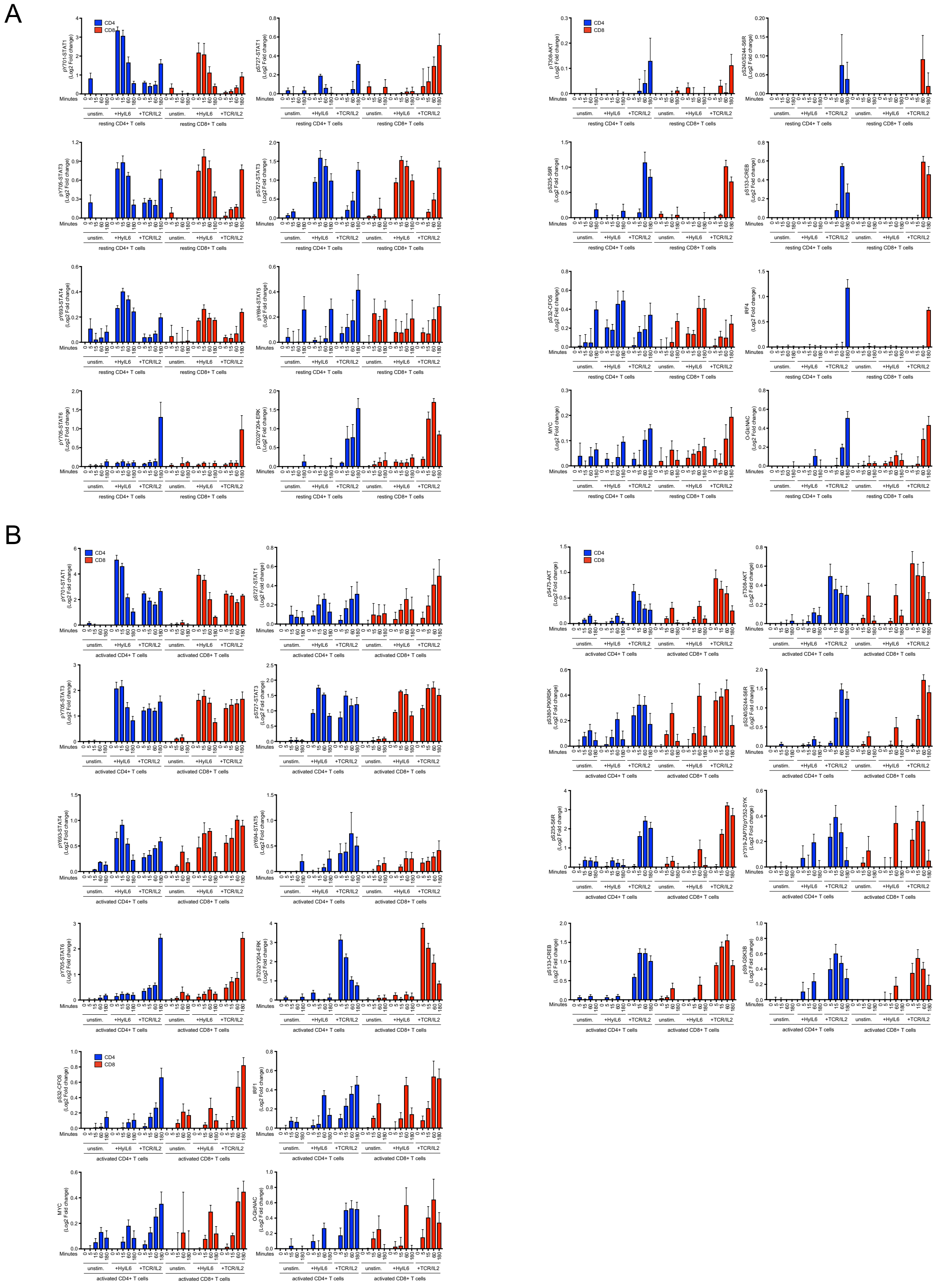

Supplemental Figure S2. Phospho-FLOW analyses of the HyIL-6 signalosome in resting and activated human primary CD4^{+} and CD8^{+} T cells: Time-course. Related to Figure 1. A) Time-course data of the changes induced by HyIL-6 in the phosphorylation state of the main signaling pathways in resting human primary CD4^{+} and CD8^{+} T cells unstimulated, treated with HyIL-6 or with anti-CD3+Interleukin-2, as shown in Figure 1C. **B)** Time-course data of the changes induced by HyIL-6 in the phosphorylation state of the main signaling pathways in activated human primary CD4^{+} and CD8^{+} T cells unstimulated, treated with HyIL-6 or with anti-CD3+Interleukin-2, as shown in Figure 1D. For all experiments data represents mean ± SEM calculated from three individual biological replicas.

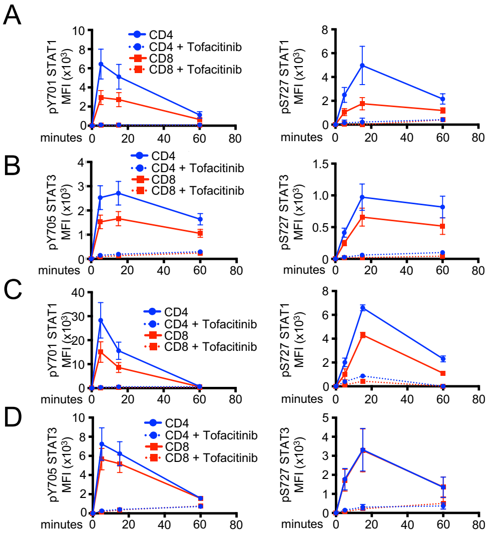

E

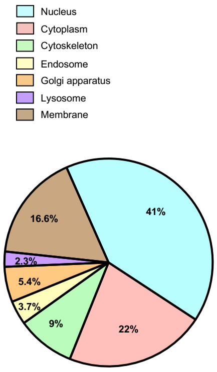

Nucleus	ADAR, ATR, ATRX, BARD1, PRDM1, C1QBP, RUNX2, CDC5L, CENCP, CENPF, CHD2, CREM, ELF2, ERF, ETV6, FOXO1, FLI1, MTOR, XRCC6, GFI1, GTF2F1, GTF2I, GTF3C2, HIST1H1E, HCFC1, HIC1, HMGA1, HNRNPH1, HNTNPK, HNRNPU, AGFG1, HSPB1, IFI16, ILF3, INCENP, IRF3, LIG1, MBP, MCM4, MECP2, MEF2C, MKI67, KMT2A, AFF1, MTF1, NACA, NCBP1, NFATC2, NPAT, NUMA1, PKNOX1, POLR2I, MED1, PPID, PPP1CC, PPP1R7, PPP1R10, PRCC, PRKAA1, PRKCD, MAPK1, MAPK3, PSMD11, PTPN2, PTPN11, RANBP2, KDM5A, RBBP7, RRLB2, RELB, RFC1, RFC2, RLF, RPS6KA1, ATXN1, SFSWAP, SP4, STAT1, STAT3, STAT4, STAT5A, STAT5B, STAT6, STK4, SUV39H1, TCF4, TCF3, TCF20, VPS72, TCOF1, TMF1, TP53BP1, TP53BP2, NR2C1, TTF1, XRCC5, ZNF8, NELFE, KAT6A, NCOA3, HIST1H4I, TRRAP, EOMES, MKNK1, NOP14, SAP30, SQSTM1, MCM3AP, PRPF4B, LIMD1, MTA1, NEMF, MTA2, STK17B, TJP2, RBM39, NCOR1, MDC1, MATR3, DDX46, HELZ, NUP153, CASP8AP2, RBM6, SF3B4, UBE4B, NDC80, MCRS1, PLK4, NUP50, PPP1R13L, ARID5A, AKAP13, ATXN2L, SPEN, KDM1A, PDS5B, ZNF609, CIC, PASK, SMG6, UBR2, NCAPH, SF3B1, ETHE1, NUP188, SRRM2, KIF4A, SUN2, NIPBL, POLDIP2, TOR1AIP1, NOCL, STAU2, FOXP1, AFF4, INVS, ZNF638, CPSF1, HP1BP3, WAC, UBR5, YTHDF2, RSF1, MPHOSPH8, ZNF280D, BCAS3, KANSL2, PHIP, RIF1, TMEM57, MIS8BP1, ZCCHC8, CDKN2AIP, CHD7, WDR11, LRIF1, CENPN, WRNIP1, EMSY, REXO1, GATAD2B, ZNF687, RANBP10, CCAR2, KMT2C, EPS15L1, RBM25, WIZ, UBE2O, ZNF106, PAPOLG, AHNAK, PHC3, AKNA, FIP1L1, TSC22D4, FAM175A, FYTTD1, PARP10, ZNF276, AGAP2, SPATA33, SSBP4, CRTC2, HIPK1, RNF169, BCL9L, NACA2
Cytoplasm	ADD3, APRT, BNIP2, CDC25B, DAB2, MTOR, GPI, IREB2, STMN1, LCP1, LIPE, MAP1A, MOV10, MYH8, PFAS, PI4KA, MAPK1, MAPK3, PSMD11, PTPN2, PTPN11, PXN, WARS, RPS6KA1, SNTB1, STAT1, STAT3, STAT4, STAT5A, STAT5B, STAT6, STK4, TALDO1, TRAF2, FXR1, MKNK1, IRS2, EIF3C, RIPK1, SYNJ1, ARHGEF7, SQSTM1, HERC1, ARHGEF2, CCP110, IFT140, FAM65B, CASP8AP2, OPTN, MPHOSPH9, PLIN3, UBE4B, GAS2L1, PLK4, LZTS1, AKAP13, AKAP11, COPE, FNBP1, KIF1B, CUL9, TAB2, CLASP2, EPB41L3, MAST2, FRMD4B, EFR3A, PASK, SMG6, ANKS1A, KIF13B, LARP1, ETHE1, MACF1, SCRIB, PRKD3, RNF19A, SIPA1L1, SACS, GPSM2, DUSP13, BIN2, YTHDF2, LIMA1, FBXW5, PPP1R12C, HAUS6, TRIT1, PANK4, DENND4C, MAP7D1, ASUN, KIZ, KIAA1217, RALGAPA2. RANBP10, UBE2O, SMAP2, MICAL1, EPS8L2, CORO7, MICALL2, CARD11, FGD3, KLC4, FAM129A, RFFL, SPATA33, FAM101B, MAST4, CCDC88C
Cytoskeleton	AKAP11, BCAS3, FRMD4B, FGD3, GPSM2, HAUS6, LIMA1, MPHOSPH9, MAP7D1, MICALL2, RELB, ARHGEF2, ADD3, CDC25B, CCP110, CENPF, CLASP2, EPB4L13, FAM65B, FNBP1, GAS2L1, HSPB1, INCENP, IFT140, INVS, KIF13B, KIF1B, KIF4A, KLC4, KIZ, LCP1, MTA1, MICAL1, MAP1A, MAST2, MACFC1, MAPK1, NUMA1, PXN, PLK4, FAM101B, RIF1, RNF19A, SIPA1L1, STMN1M, SNTB1
Endosome	MICALL1, MICALL2, RAB10, RAPGEF1, SUN2, TBCD1D5, ATG9A, FAM21A, FAM21C, INPP5F, OPTN, OSBPL11, OSBPL9, PLIN3, RFFL, SQSTM1, SPPL2B, SNX3, VAMP7
Golgi apparatus	ATR, LIG1, ERF, FGD3, HERC1, LRBA, MPHOSPH9, PASK, RAB10, ARHGEF2, SEC16A, TMF1, ACSL3, COPE, CORO7, CLASP2, KAT6A, HLA-A, MACF1, MAPK1, MAPK3, OPTN, OSBPL11, OSBPL9, PLIN3, PARP10, TRRAP, VAMP7
Lysosome	AHNAK, DAB2, WDR11, CLCN7, C18ORF8, HM13, IGF2R, MTOR, SPPL2B, SLC12A4, STX8, VAMP7
Membrane	CNP, AKAP13, AHNAK, AKNA, AGAP2, CD300A, CD5, DDX46, FXR1, GIGYF2, HERC1, LRBA, LARP1, MPHOSPH9, NDC80, NOP14, RANBP2, WDR11, WRNIP1, XRCC5, XRCC6, ACSL3, ADD3, ADAR, AVEN, ATXNL2L, ATG9A, CDC5L, CLCN7, CCDC77, CCDC88B, C1QBP, CORO7, CLASP2, DOCK10, EPS15L1, EIF4EBP3, FAM129A, GTF2I, GPI, HELZ, HNRNPH1, HNRNPK, HNRNPU, HM13, HIST1H4I, HCFC1, IGF2R, ITGAL, IFI16, ILF3, INVS, KIF4A, LTK, LSP1, HLA-A, MKI67, MATR3, MTOR, MED1, MTA2, MCM4, NCAPH, NCOR1, NUP188, PLIN3, PI4K1, PIK3R1, PAPOLG, PSMD11, PRKCD, PTPRC, RPL4, RFFL, SEMA4C, SPPL2B, SLC12A4, SLC4A3, SORCS1, STMN1, STAU2, STX4, UBR5, VAMP7, ZNF106

Supplemental Figure S3. Effect of Tofacitinib in the HyIL-6-induction of Tyr and Ser phosphorylation in STAT3 and STAT1 in resting and activated primary human CD4$^+$ and CD8$^+$ T cells (A-D) and Proteins regulated by HyIL-6 in human primary CD4$^+$ Th-1 cells (E). Related to Figures 1 and 2. A) Time-course of STAT1 Tyr701 (left panel) and Ser727 (right panel) phosphorylation induced by IL-6 stimulation in the presence (dash line) or absence (solid line) of 2µM Tofacitinib in resting primary human CD4$^+$ and CD8$^+$ T cells. **B)** Time-course of STAT3 Tyr705 (left panel) and Ser727 (right panel) phosphorylation induced by IL-6 stimulation in the presence (dash line) or absence (solid line) of 2µM Tofacitinib in resting primary human CD4$^+$ and CD8$^+$ T cells. **C)** Time-course of STAT1 Tyr701 (left panel) and Ser727 (right panel) phosphorylation induced by IL-6 stimulation in the presence (dash line) or absence (solid line) of 2µM Tofacitinib in activated primary human CD4$^+$ and CD8$^+$ T cells. **D)** Time-course of STAT3 Tyr705 (left panel) and Ser727 (right panel) phosphorylation induced by IL-6 stimulation in the presence (dash line) or absence (solid line) of 2µM Tofacitinib in activated primary human CD4$^+$ and CD8$^+$ T cells. For all experiments quantitative data was calculated from three individual biological replicas. Error bars show mean ± SEM. **E)** The scheme shows the cellular location (See Supplementary Figure 5) and molecular function of the proteins regulated by phosphorylation in response to HyIL-6 stimulation in human primary CD4$^+$ Th-1 cells as determined by DAVID analysis. Refer also to Supplementary Figure 4.

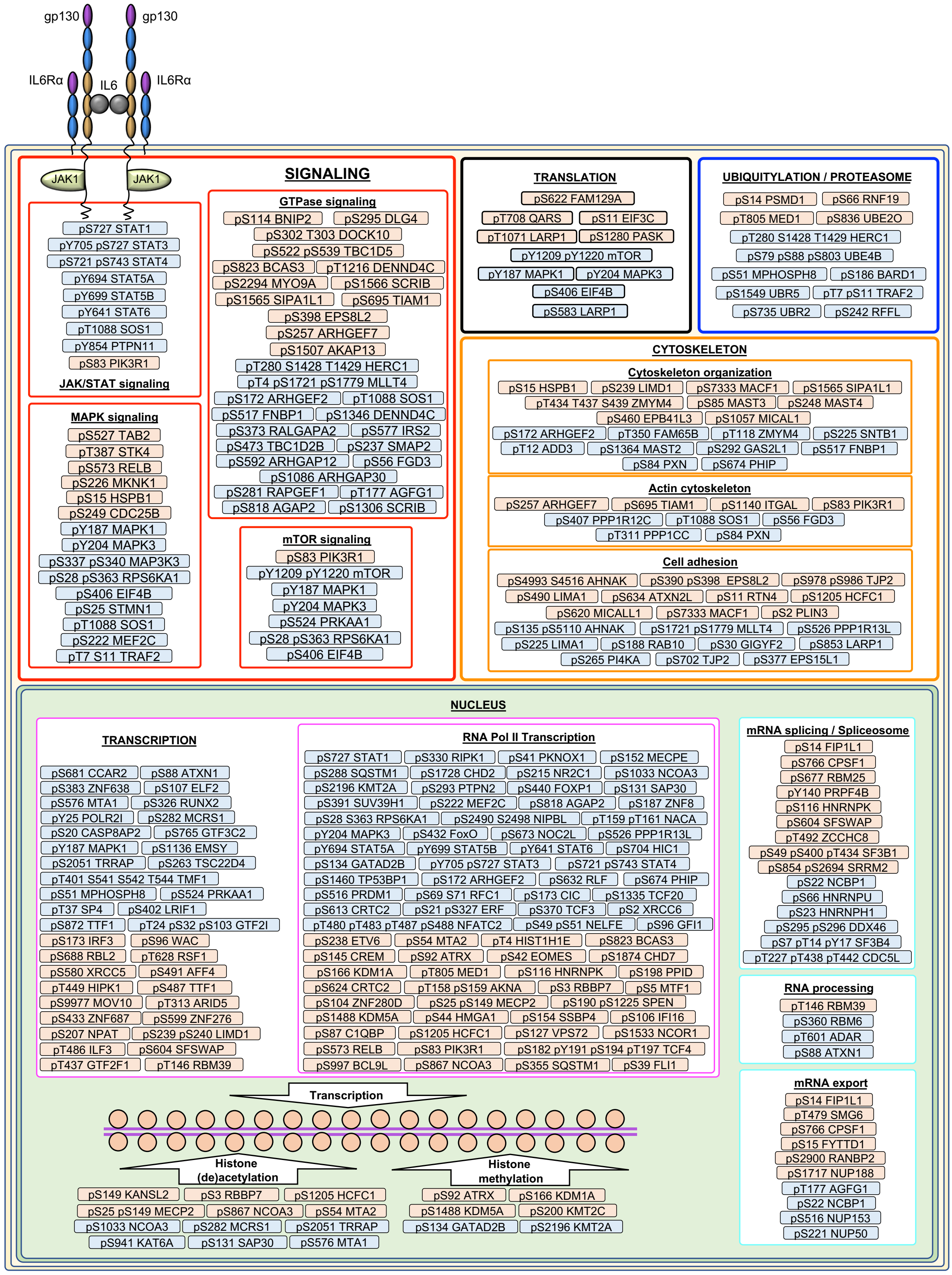

Supplemental Figure S4. Cellular location of the proteins regulated by HyIL-6 in primary human Th-1 cells. Related to Figure 2. GO analysis of the cellular location of the proteins regulated by phosphorylation in human primary CD4^{+} Th-1 cells in response to HyIL-6 alongside with a table showing the different proteins identified in our study and their subcellular location as inferred from our GO analysis.

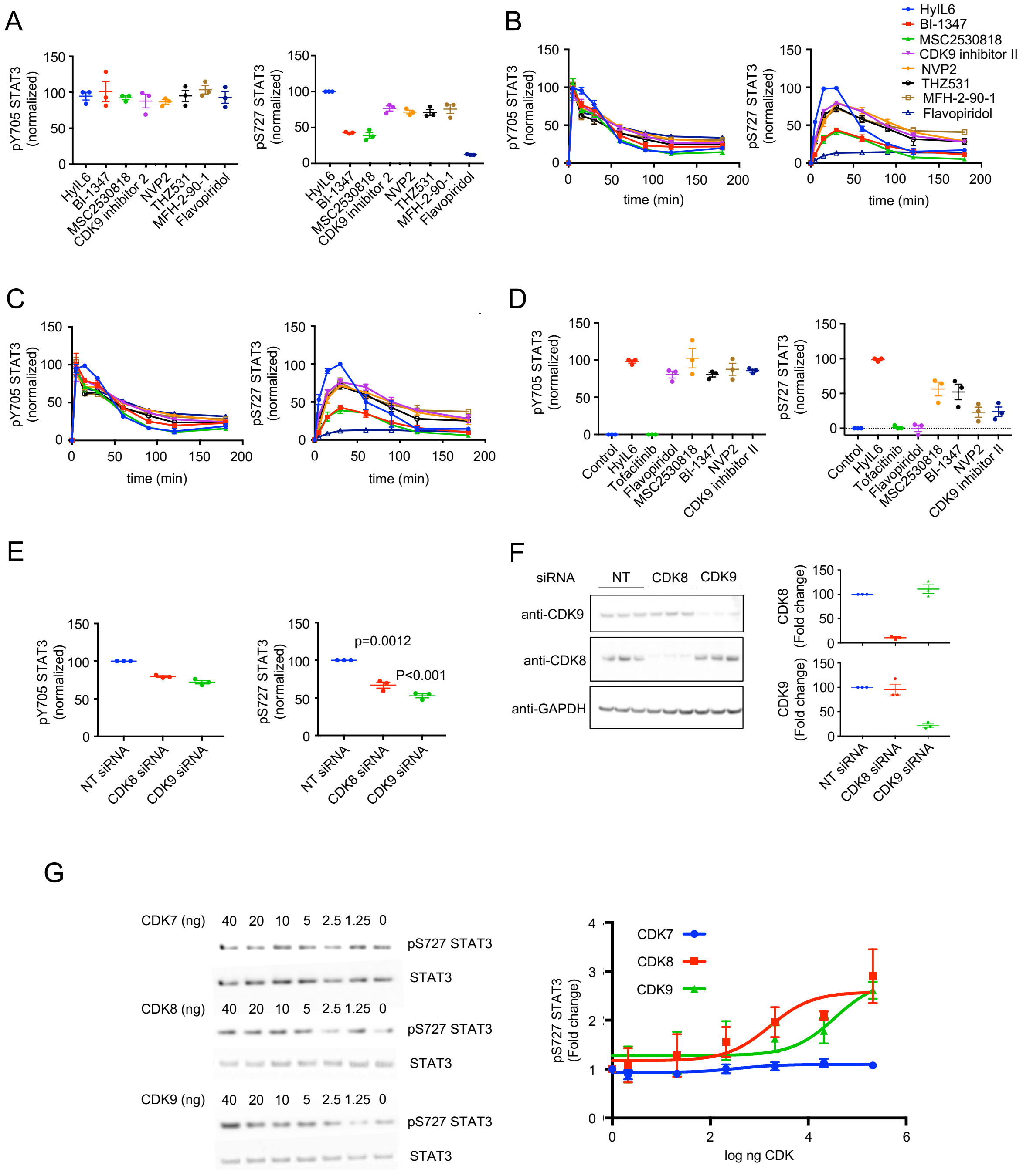

Supplemental Figure S5. Role of CDKs in the regulation of Ser727 STAT3 and STAT1 phosphorylation. Related to Figures 3 and 4. A) Effect of different CDK inhibitors on the STAT3 Tyr705 (left panel) and STAT3 Ser727 (right panel) phosphorylation induced by HyIL-6 in human primary $CD8^{+}$ T cells. **B)** Kinetics of the HyIL-6-induced STAT3 Tyr705 (left panel) and Ser727 (right panel) phosphorylation in the presence of different CDK inhibitors in human primary $CD4^{+}$ T cells. **C)** Kinetics of the HyIL-6-induced STAT3 Tyr705 (left panel) and Ser727 (right panel) phosphorylation in the presence of different CDK inhibitors in HEK293T cells. **D)** Effect of different CDK inhibitors on the STAT3 Tyr705 (left panel) and STAT3 Ser727 (right panel) phosphorylation induced by HyIL-6 in HEK293T cells. **E)** Effect of knocking-down CDK8 or CDK9 in the STAT3 Tyr705 (left panel) and STAT3 Ser727 (right panel) phosphorylation induced by HyIL-6 in HEK293T cells. **F)** Immunoblot analysis of the knocking-down of CDK8 or CDK9 in HEK293T cells. **G)** In vitro phosphorylation of STAT3 Ser727 by varying amounts of CDKs. Quantitation showing the dose response effect is shown alongside. For all experiments quantitative data was calculated from three individual biological replicas. Error bars show mean ± SEM.

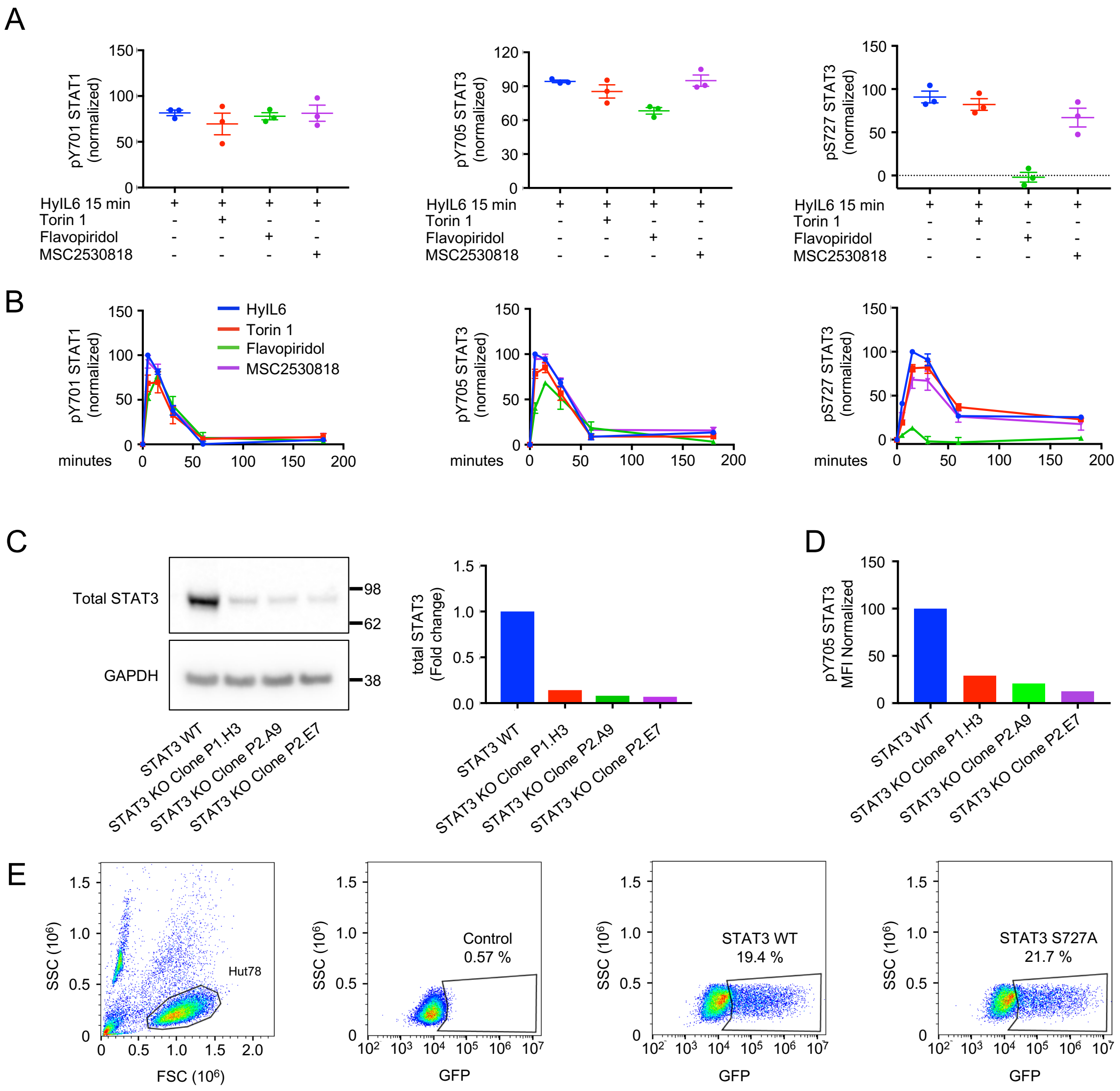

Supplemental Figure S6. Regulation of Ser727 STAT3 phosphorylation in Hut78 cells. Related to Figure 4. A) Effect of Torin1, Flavopiridol or MSC2530818 on the STAT1 Tyr701 (left panel), STAT3 Tyr705 (middle panel) and STAT3 Ser727 (right panel) phosphorylation induced by HyIL-6 in human primary $CD4^+$ T cells as measured by phosphor-FLOW. **B)** Time-course of STAT1 Tyr701 (left panel), STAT3 Tyr705 (middle panel) and STAT3 Ser727 (right panel) phosphorylation induced by HyIL-6 in human primary $CD4^+$ T cells in the presence or absence of Torin1, Flavopiridol or MSC2530818 as measured by phosphor-FLOW. **C)** STAT3 immunoblot of Hut78 WT cells vs Crispr/CAS9 generated STAT3 KnDs Hut78 cells. Quantitation of the levels of STAT3 are shown in the graph. **D)** FACS analysis of the level of STAT3 Tyr705 phosphorylation in Hut78 WT and the different STAT3 KnDs Hut78 cell lines upon 15 min HyIL-6 stimulation. **E)** FACS analysis of the expression of STAT3 WT-GFP or STAT3 S727A-GFP recombinant proteins in Hut78 STAT3 KnDs electroporated with pLV-CMV-GFPSpark plasmid. For experiments **A-B** quantitative data shows normalized data ± SEM of three individual biological replicas.

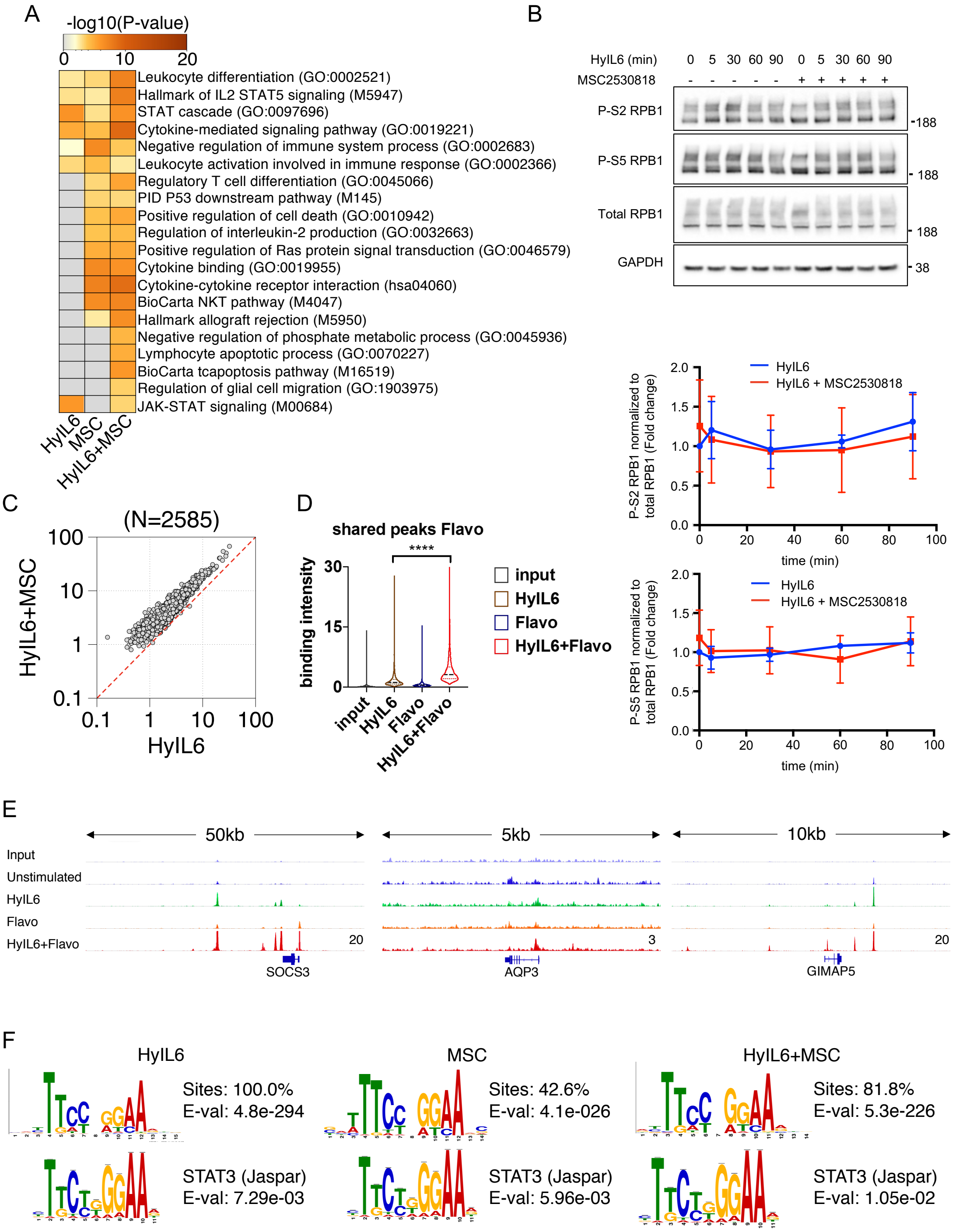

Supplemental Figure S7. CDK8 fine-tunes STAT3 transcriptional program. Related to Figure 6. A) Pathway analysis of differently expressed genes before and after HyIL-6, MSC or HyIL-6+MSC stimulation using Metascape (Zhou et al., 2019). Top 20 pathways are shown. **B)** Immunoblot analysis of the Ser2 and Ser5 phosphorylation state of RPB1 in Th-1 cells treated or untreated with MSC2530818 upon HyIL-6 stimulation. Quantitative data shows normalized data ± SEM of three individual biological replicas. **C)** Scatter plot comparing the mean STAT3 binding intensity in n=2585 STAT3 bound regions between HyIL-6+MSC versus HyIL-6. **D**) Violin plot showing the mean STAT3 binding intensity in n= 4359 STAT3 bound regions across different stimulations. Peaks are identified by comparing HyIL-6+Flavo stimulation and input. P-values are determined by two-tailed Wilcoxon test (**** p<0.0001). **E**) Representative loci showing STAT3 binding across different stimulations. The height of the tracks are indicated at bottom right corner of the plots. **F)** Shown are the most significant de novo motifs identified in STAT3 bound regions after HyIL6, MSC or HyIL6+MSC stimulation (top) and the matched STAT3 motif (bottom) from JASPAR database using TOMTOM.
